# How Naive T-Cell Clone Counts Are Shaped By Heterogeneous Thymic Output and Homeostatic Proliferation

**DOI:** 10.3389/fimmu.2021.735135

**Published:** 2022-02-17

**Authors:** Renaud Dessalles, Yunbei Pan, Mingtao Xia, Davide Maestrini, Maria R. D’Orsogna, Tom Chou

**Affiliations:** ^1^ Department of Computational Medicine, University of California at Los Angeles (UCLA), Los Angeles, CA, United States; ^2^ Department of Mathematics, California State University at Northridge, Los Angeles, CA, United States; ^3^ Department of Mathematics, University of California at Los Angeles (UCLA), Los Angeles, CA, United States

**Keywords:** naive T cells, T-cell receptor, repertoire diversity, clone-count distributions, mathematical modeling, immigration-proliferation model, heterogeneity

## Abstract

The specificity of T cells is that each T cell has only one T cell receptor (TCR). A T cell clone represents a collection of T cells with the same TCR sequence. Thus, the number of different T cell clones in an organism reflects the number of different T cell receptors (TCRs) that arise from recombination of the V(D)J gene segments during T cell development in the thymus. TCR diversity and more specifically, the clone abundance distribution, are important factors in immune functions. Specific recombination patterns occur more frequently than others while subsequent interactions between TCRs and self-antigens are known to trigger proliferation and sustain naive T cell survival. These processes are TCR-dependent, leading to clone-dependent thymic export and naive T cell proliferation rates. We describe the heterogeneous steady-state population of naive T cells (those that have not yet been antigenically triggered) by using a mean-field model of a regulated birth-death-immigration process. After accounting for random sampling, we investigate how TCR-dependent heterogeneities in immigration and proliferation rates affect the shape of clone abundance distributions (the number of different clones that are represented by a specific number of cells, or “clone counts”). By using reasonable physiological parameter values and fitting predicted clone counts to experimentally sampled clone abundances, we show that realistic levels of heterogeneity in immigration rates cause very little change to predicted clone-counts, but that modest heterogeneity in proliferation rates can generate the observed clone abundances. Our analysis provides constraints among physiological parameters that are necessary to yield predictions that qualitatively match the data. Assumptions of the model and potentially other important mechanistic factors are discussed.

## Introduction

Naive T cells play a crucial role in the immune system’s response to pathogens, tumors, and other infectious agents. These cells are produced in the bone marrow, mature in the thymus, circulate through the blood, and migrate to the lymph nodes where they may be presented with different antigen proteins from various pathogens. Naive T cells mature in the thymus where the so-called V, D, and J segments of genes that code T cell receptors undergo rearrangement. Most T cell receptors (TCRs) are comprised of an alpha chain and a beta chain that are formed after VJ segment and VDJ segment recombination, respectively. The number of possible TCR gene sequences is extremely large, but while recombination is a nearly random process, not all TCRs are formed with the same probability.

The unique receptors expressed on the cell surface of circulating TCRs enable them to recognize specific antigens; well-known examples include the naive forms of helper T cells (CD4+) and cytotoxic T cells (CD8+). The set of naive T cells that express the same TCR are said to belong to the same T cell clone. Upon encountering the antigens that activate their TCRs, naive T cells turn into effector cells that assist in eliminating infected cells. Effector cells die after pathogen clearance, but some develop into memory T cells. Because of the large number of unknown pathogens, TCR clonal diversity is a key factor for mounting an effective immune response. Recent studies also reveal that human TCR clonal diversity is implicated in healthy aging, neonatal immunity, vaccination response and T cell reconstitution following haematopoietic stem cell transplantation (1, 2). Despite the central role of the naive T cell pool in host defense, and broadly speaking in health and disease, TCR diversity is difficult to quantify. For example, the human body hosts a large repertoire of T cell clones, however the actual distribution of clone sizes is not precisely known (3). Only recently have experimental and theoretical efforts been devoted to understanding the mechanistic origins of TCR diversity (4–9). The goal of this work is to formulate a realistic mathematical model that incorporates heterogeneity in naive T cell generation and reproduction. Model predictions are compared with T cell clone data to estimate reasonable and realistic parameter values.

One way to describe the TCR repertoire is by tallying the population *n_i_* of T cells carrying receptor *i*. Another is to use the clone abundance distribution or “clone count” that measures the number of distinct clones composed of exactly *k* T cells, ${\hat{c}}_{k}:=\Sigma_{i=1}^{\infty }$ 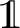
(*n_i_, k*), where the indicator function 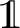
(*n*, *k*) = 1 if *n* = *k* and 0 otherwise. Clone counts ${\hat{c}}_{k}$ do not carry TCR identity information as *n_i_* does, however, they can be used to construct other summary indices for T cell diversity such as Shannon’s entropy, Simpson’s index, or the whole population richness $\hat{C}:=\Sigma_{k=1}^{\infty }{\hat{c}}_{k}$ (10).

Clone counts ${\hat{c}}_{k}$ and the total number of circulating naive T cells are difficult to measure in humans. Nonetheless, high-throughput DNA sequencing on samples of peripheral blood containing T cells (11–14) have provided some insight into TCR diversity. A commonly invoked model is that clone counts ${\hat{c}}_{k}$ exhibit a power-law distribution (4, 12, 15–17) in the clone abundance *k*. Several models have been developed to explain observed features of clone counts (3, 4, 15, 18, 19), including the apparent power-law behavior. One proposal is that T cells in different clones have TCRs that have different affinities for self-ligands that are necessary for peripheral proliferation (4–6), leading to clone specific replication rates. An alternative hypothesis (7) is that specific TCR sequences are more likely to arise in the V(D)J recombination process in the thymus (20) leading to a higher probability that these TCRs are produced. De Greef et al. (7) estimated the probability of production of a given TCR sequence by using the Inference and Generation of Repertoires (IGoR) simulation tool that quantitatively characterizes the statistics of receptor generation from both cDNA and gDNA data (20).

**Figure 1:**
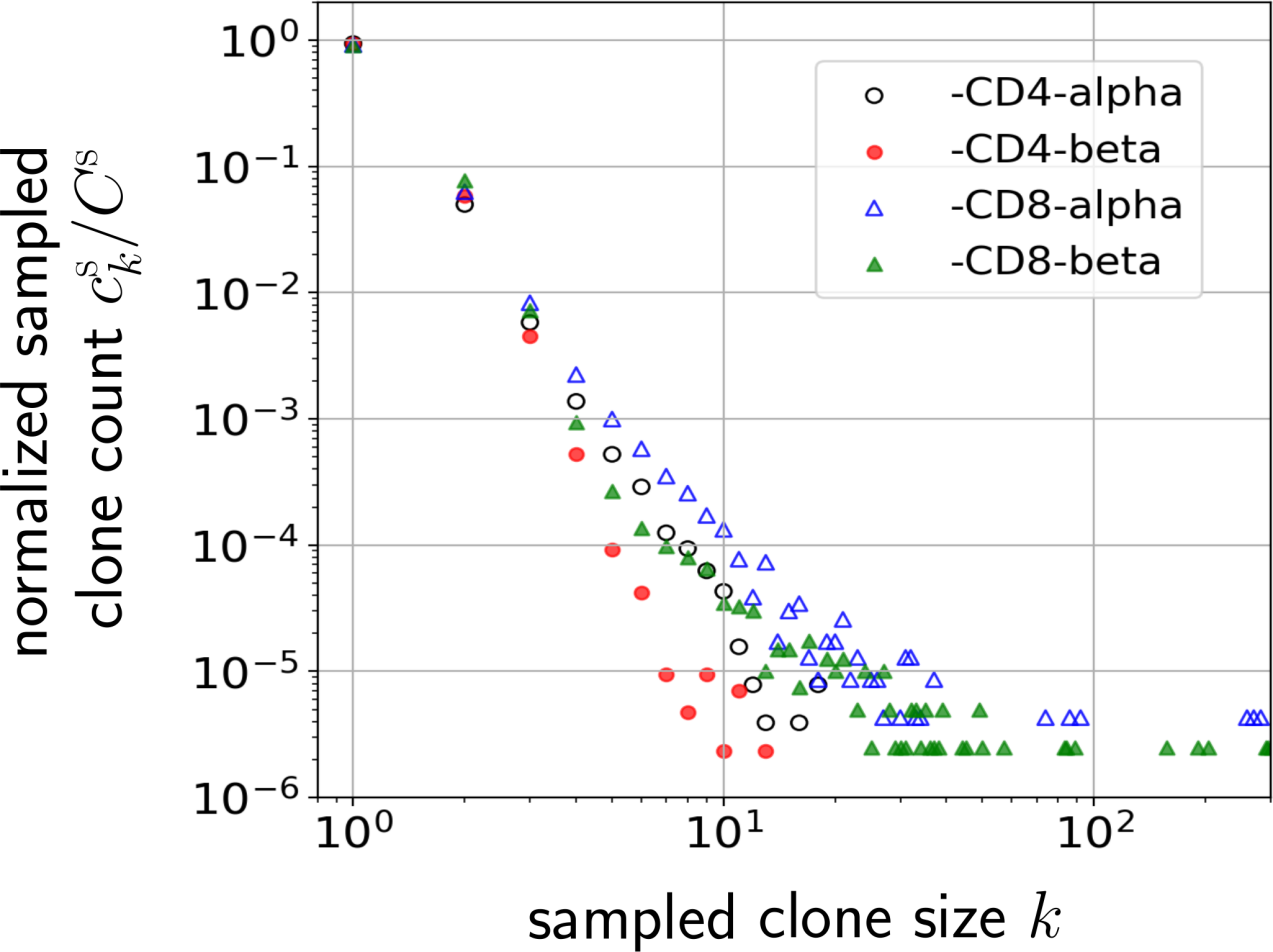
Normalized naive T cell clone count data from one patient in Oakes et al. (12) plotted on a log-log scale. Values of the normalized clone counts along the vertical axis are the average of three samples among CD4 and CD8 cell subgroups. Clones are defined by different nucleotide sequences associated with different alpha or beta chains of the TCR.

Although power-law models have been motivated, this behavior has been observed across only about two decades of clone sizes *k*, as shown in **Figure 1**. Moreover, the above models have not systematically incorporated and compared heterogeneity in both immigration and replication rates, and/or fitted models to measured TCR clone abundance distributions. Finally, some of them have not taken into account subsampling in measurements, which will affect the predicted clone counts, especially for small clone sizes *k* which can be missed in small samples. In this paper, we analyze the effects of heterogeneity and sampling within a dynamic mean-field model based on a stochastic clone-dependent birth-death-immigration (BDI) process that includes (i) immigration representing the arrival of new clones from the thymus, (ii) birth during homeostatic proliferation of naive T cells that yield newborn naive T cells with the same TCR as their parent, and (iii) death representing cell apoptosis  (10). We also include a regulating “carrying capacity” mechanism through a total population-dependent death rate which may represent the global competition for cytokines, such as Interleukin-7 (21–25), needed for naive T cell survival and homeostasis (26, 27). Since these cytokine signals are TCR-independent, the regulatory interaction, which ensures a finite homeostatic naive T cell population, is clone-independent (23).

We derive analytic expressions for the steady state clone counts in the entire organism and show that the predicted distributions are negative binomials. However, since T cell clone populations are measured in small blood subsamples extracted from an organism, we modify our predictions to include the effects of random subsampling and find that the negative binomial structure is preserved. Finally, the subsampled prediction will be averaged over distributions of TCR generation (thymic output) and homeostatic proliferation rates. The distribution of TCR generation rates are extracted from new computational tools: Inference and Generation of Repertoires (IGoR) (20) and Optimized Likelihood estimate of immunoGlobulin Amino-acid sequences (OLGA) (28). Since there are no equivalent tools that measure proliferation rates, we will assume simple functions for the distribution of homeostatic proliferation rates. These model-derived results depend on the rate parameters of the model and the hyperparameters defining the probability distributions over these T cell production and proliferation rates (see **Table 1**).

**Table 1:** Model parameters *θ* and hyperparameters *θ*_0_.

(Hyper) Parameters	definition
*α ∈ ℝ^+^*	naive T cell production rate
$\bar{\alpha }\in {\mathbb{R}}^{+}$	mean production rate across all possible *Q* TCRs
*r ∈* [0, *R*]	naive T cell proliferation rate
$\bar{r}\in {\mathbb{R}}^{+}$	mean proliferation rate across all possible *Q* TCRs
*R ∈* ℝ^+^	maximum proliferation rate of all possible *Q* TCRs
*w ∈* [0, 1]	dimensionless width of box distribution of *r*
*µ* ^∗^ > *R*	naive T cell death rate at steady state
*η ∈* [0,1]	blood subsampling fraction

The dimensional parameters associated with our mechanistic population model. Hyperparameters such as $\bar{\alpha }$ , r, R, w define the probability distribution or heterogeneity in the underling rate parameters $\bar{\alpha }$ and r. In our analyses, we typically nondimensionalize by normalizing all rates by R, the maximum proliferation rate across all clones.

Our results are then compared to the data shown in **Figure 1** and used to estimate hyperparameters associated with the heterogeneity in the TCR-specific immigration and proliferation rates. Specifically, we quantify how the width of a simple uniform proliferation rate distribution and the heterogeneity of immigration rates from a generative model affect the predicted clone counts. Our analysis explicitly shows that within reasonable physiological parameter ranges, heterogeneity in the thymic immigration rate cannot significantly change clone count distributions. However, clone counts *are* sensitive to heterogeneity in T cell proliferation rates. Thus, different levels of heterogeneity in proliferation rates can give rise to qualitatively different clone count distributions. This finding of the dominance of proliferation in shaping clone count distributions is consistent with the observation that in older humans with severely reduced thymic output a broad clone count distribution is still maintained (9, 29).

## Materials and Methods

To understand the observed clone counts, we focus on the clone count distribution ${\hat{c}}_{k}$ associated only with naive T cells, the first type of cells produced by the thymus that have not yet been activated by any antigen. Antigen-mediated activation initiates a largely irreversible cascade of differentiation into effector and memory T cells that we can subsume into a death rate. Thus, we limit our analysis to birth, death, and immigration within the naive T cell compartment. Here, we first present the mathematical framework of the BDI process to provide an initial qualitative understanding for clone counts.

### Heterogeneous Birth-Death-Immigration Model

**Figure 2:**
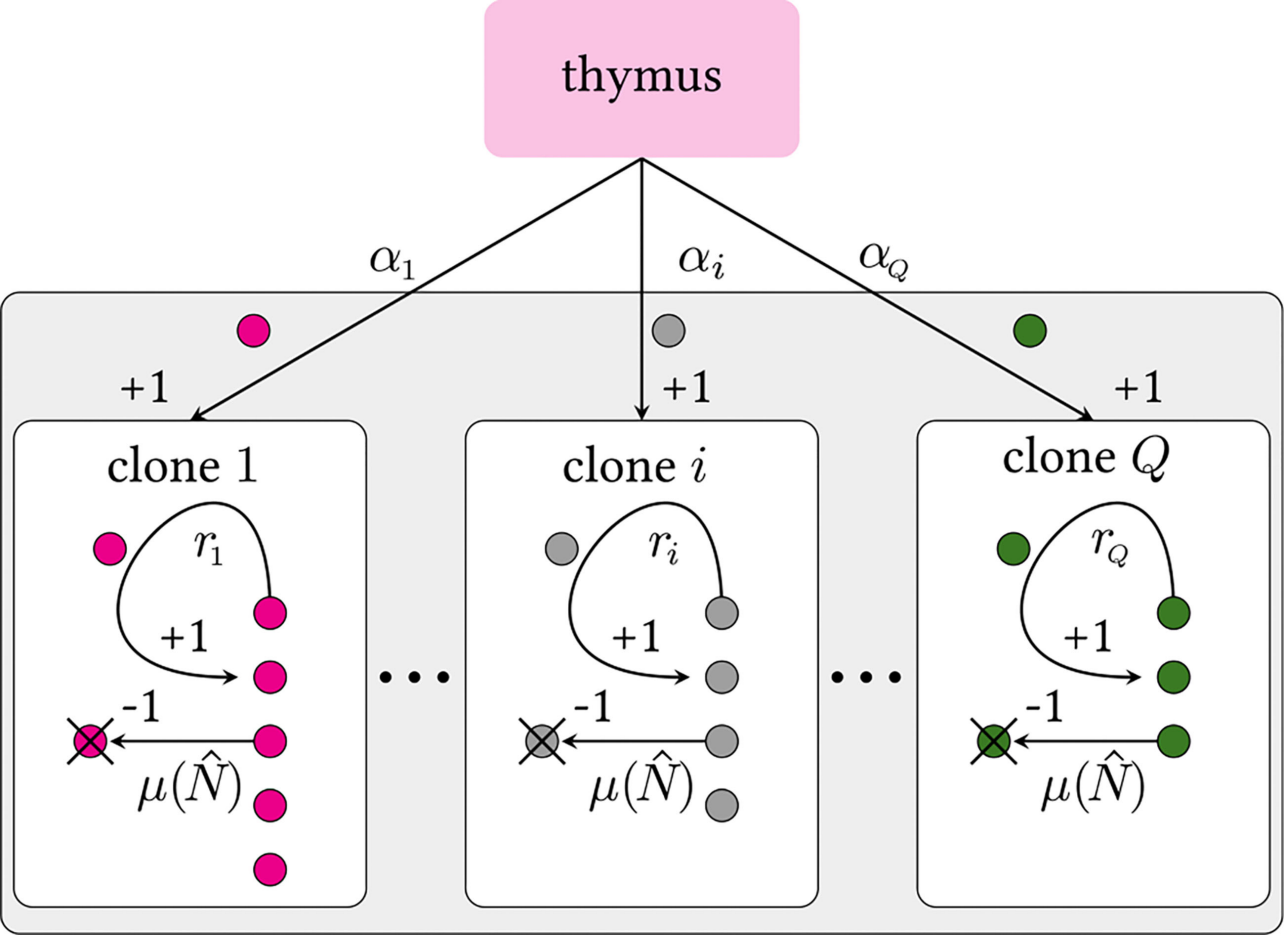
Schematic of a multiclone birth-death-immigration process. Clones are defined by distinct TCR sequences *i*. Each clone carries its own thymic output and peripheral proliferation rates, *α_i_* and *r_i_*, respectively. We assume all clones have the same population-dependent death rate *μ*(), where is the total number of cells in the organism that influence the death rate. Since *Q* ≫ 1, we impose a continuous distribution over the rates *α* and *r*. Theoretically, there may be *Q* **≳** 10^15^ (6) or more (30, 31) possible viable V(D)J recombinations. The actual, effective number of different selected TCRs sequences is expected to be much less since extremely low probability sequences may never be formed during the organism’s lifetime. A strict lower bound on *Q* is the actual number of distinct clones *Ĉ* in an entire organism [*Ĉ* ∼ 10^6^ – 10^8^ for humans (1, 6, 32–34)].

The multiclone BDI process is depicted in **Figure 2**. We define *Q* to be the theoretical number of all possible functional naive T cell receptor clones that can be generated by V(D)J recombination in the thymus which is estimated to be *Q* ~ 10^13^ – 10^18^ (6, 28). As we will later show, results of our model will not depend on the explicit value of *Q* as long as *Q* ≫ 1. Due to naive T cell death or removal from the sampling-accessible pool, not all possible clone types will be presented in the organism, so we denote the number of clones actually present in the body (or “richness”) by $\hat{C}\ll Q$ , where estimates of $\hat{C}$ range from ~ 10^6^ – 10^8^ in mice and humans (1, 6, 32, 33, 35, 36).

Although naive T cells are difficult to distinguish from the entire T cell population, the total number of naive T cells (across all clones present) in humans has been estimated to be about $\hat{N}∼10^{11}$ . Circulating naive T cells number approximately 10^9^ (37) but can exchange, at different time scales, with those that reside in peripheral tissue, which may carry their own proliferation and death rates. The *effective* pool that is ultimately sampled is thus difficult to estimate, but measurements show that the theoretical number of different clones is much larger than the total number of naive T cells, which is in turn much greater that the total number of different T cell clones actually in the body $\left(Q\gg \hat{N}\gg \hat{C}\right)$ . Regardless of the precise values of the discrete quantities $Q,\hat{N},\hat{C}$ , they are related to the discrete clone counts ${\hat{c}}_{k}$ via


(1) $$\hat{C}=\sum_{k\geq 1}{\hat{c}}_{k}\ll Q\text{ and }\hat{N}=\sum_{k\geq 1}k{\hat{c}}_{k}.$$

As depicted in **Figure 2**, each distinct clone *i* (with 1 ≤ *i* ≤ *Q*) is characterized by an immigration rate *α_i_* and a per cell replication rate *r_i_*. The immigration rate *α_i_* is clone-specific because it depends on the preferential V(D)J recombination process; the replication rate *r_i_* is also clone-specific due to the different interactions with self-peptides that trigger proliferation. Since both the numbers of theoretically possible (Q≫1) and observed $\left(\hat{C}\gg 1\right)$ clones are extremely large, we can define a continuous, normalized probability density π(*α*, *r*) from which immigration and proliferation rates *α* and *r* of a randomly chosen clone are drawn. This means that the probability that a randomly chosen clone has an immigration rate between *α* and *α* + d*α* and replication rate between *r* and *r* + d*r* is *π*(*α*, *r*)d*α*d*r*, and $\int_{0}^{\infty }\text{d}\alpha \int_{0}^{\infty }\text{d}r\pi \left(\alpha ,r\right)=1$ .

Since *Q* is finite and countable, there will exist maximum values *A* and *R* for the immigration and proliferation rates, respectively, such that *π*(*α, r*) = 0 for *α > A* or *r > R*. In the BDI process, the upper bound *R* on the proliferation rate prevents unbounded numbers of naive T cells and is necessary for a self-consistent solution. The heterogeneity in the immigration and replication rates allows us to go beyond typical “neutral” BDI models, where both rates are fixed to a specific value for all clones, *α_i_ = α* and *r_i_ = r* for all *i*.

Finally, we assume the per cell death rate $\mu (\hat{N})$ is clone-independent but a function of the total population $\hat{N}$ . This dependence represents the competition among all naive T cells for a common resource (such as cytokines), which effectively imposes a carrying capacity on the population (24, 31, 38). The specific form of the regulation will not qualitatively affect our findings since we will ultimately be interested in only its value *μ*(*N* ^∗^) *≡ μ* ^∗^ at the mean steady state population *N* ^∗^.

### Mean-Field Approximation of the BDI Process

The exact steady-state probabilities of configurations of the discrete abundances ${\hat{c}}_{k}$ for a fully stochastic neutral BDI model with regulated death rate $\mu (\hat{N})$ were recently derived (10). In Dessalles et al. (10) exact results were derived for the steady-state probability $P\left({\hat{c}}_{1},{\hat{c}}_{2},\ldots ,{\hat{c}}_{k}\right)$ under uniform immigration, proliferation, and death rates α, r, and μ, respectively. The significant contribution of this paper is that we go beyond the neutral model (equal immigration, proliferation, and death rates for all clones) by allowing for heterogeneous distributions of these rates. To incorporate TCR-dependent immigration and replication rates in a non-neutral model, we must consider distinct values of *α_i_* and *r_i_* for each clone *i*. In this case, an analytic solution for the probability distribution over ${\hat{c}}_{k}$ , even at steady state, cannot be expressed in an explicit form. However, since the effective number of naive T cells ( $\hat{N}∼10^{9}-10^{11}$ (35)) is large, we can exploit a mean-field approximation to the non-neutral BDI model and derive expressions for the mean values of the discrete clone counts ${\hat{c}}_{k}$ . We will show later that under realistic parameter regimes, the mean-field approximation is quantitatively accurate. Breakdown of the mean field approximation has been carefully analyzed in other studies (39).

#### i) Deterministic Approximation for the Total Population and the Effective Death Rate

To implement the mean-field approximation in the presence of a general regulated death rate $\mu (\hat{N})$ , we start by writing the deterministic, “mass-action” ODE for the mean number of cells *n_α,r_* (*t*) with a realized immigration rate *α* and proliferation rate *r* in a BDI process


(2) $$\frac{\text{d}n_{\alpha ,r}(t)}{\text{d}t}=\alpha +rn_{\alpha ,r}(t)-\mu (N(t))n_{\alpha ,r}(t).$$

Next, we define and exploit the density of realized values of *α* and *r*. Since *Q* ≫ 1, the number of TCRs that are associated with immigration rate between *α* and *α* + d*α* and a replication rate between *r* and *r* + d*r* is denoted *Qπ*(*α, r*)d*α*d*r*, where *π*(*α, r*) is a normalized density that describes how these realized values of *α* and *r* are distributed. Our model for the total mean number *N*(*t*) of naive T cells can then be estimated as a weighted integral over all *n_α,r_* (*t*)


(3) $$N(t)=Q\int_{0}^{A}\text{d}\alpha \int_{0}^{R}\text{d}r\;n_{\alpha ,r}(t)\pi (\alpha ,r).$$

Note that the limits of the integration above can equivalently be taken as *A, R*→∞ as long as *π*(*α, r*) = 0 when *α > A* or *r > R*. At steady-state, the solution to Eq. 2 can be simply expressed as


(4) $$n_{\alpha ,r}^{*}=\frac{\alpha }{\mu (N^{*})-r}$$

in which *N* ^∗^ is the predicted steady-state value of *N*(*t*) as *t* → ∞. Thus, upon weighting Eq. 4 over all possible values of *α* and *r*, we find


(5) $$N^{*}=Q\int_{0}^{R}\text{d}r\int_{0}^{\infty }\text{d}\alpha \frac{\alpha \pi \left(\alpha ,r\right)}{\mu \left(N^{*}\right)-r’}$$

a self-consistent equation for *N* ^∗^ which depends implicitly on the parameters that define the distribution *π*(*α, r*). Eq. 5 clearly shows why a finite cutoff *π*(*α, r > R*) = 0, *R* < *μ*(*N* ^∗^) is required since the integral diverges if *π*(*α, r ≥ μ*(*N* ^∗^)) > 0. However, as long as *π*(*α, r*) decays faster than 1/*α* ^2^, the *α*-integration converges with an explicit cutoff *A*.

We will first assume that *α* and *r* are uncorrelated and that the distribution factorises: *π*(*α,r*) = *π_α_* (*α*)*π_r_* (*r*). Then, the self-consistent effective steady state death rate *μ* ^∗^ *≡ μ*(*N* ^∗^) depends only on the combination


$$\frac{N^{*}}{\left(\bar{\alpha }Q\right)}=\int_{0}^{R}\frac{\text{d}r\pi_{r}\left(r\right)}{\left(\mu^{*}-r\right)},$$

where


$$\bar{\alpha }\equiv \int_{0}^{A}\alpha \pi_{\alpha }\left(\alpha \right)\text{d}\alpha$$

is the mean immigration rate across all possible clones. To simplify subsequent notation, we normalize all rates by the maximum proliferation rate *R*. To avoid population blow-up, we impose that the maximum proliferation is smaller than the steady-state death rate *R < μ* ^∗^. By measuring time in units of 1/*R*, we redefine *r/R* → *r* ≤ 1, *α/R* → *α*, $\bar{\alpha }/R\to \bar{\alpha }$ , *μ* ^∗^/*R* → *μ* ^∗^, and *R* ^2^ *π*(*α, r*) → *π*(*α, r*) so that these quantities are now dimensionless, unless otherwise explicitly stated. The steady-state self-consistent condition becomes


(6) $$\frac{N^{*}}{\bar{\alpha }Q}\equiv \frac{\lambda }{\bar{\alpha }}=\int_{0}^{1}\text{d}r\frac{\pi_{r}\left(r\right)}{\mu^{*}-r}.$$

Since the effective *Q* is a large, uncertain number, we parameterize our model in terms of *λ* ≡ *N* ^∗^/*Q*, the total steady state naive T cell population normalized by the total possible number of clones *Q*. It is sometimes deemed a measure of the “coverage” of the entire repertoire (6). Values of *N* ^∗^ and *Q* that are consistent with measurements and physiologic expectations give *λ* ≪ 1. Once $\lambda /\bar{\alpha }$ and *π_r_* (*r*) are estimated, we can self-consistently determine *μ* ^∗^ from Eq. 6. Besides $\lambda /\bar{\alpha }$ , the self-consistent value of *μ* ^∗^ will also depend on the function *π_r_* (*r*). Note from the form of Eq. 6, the self-consistent *μ* ^∗^ is inversely related to *λ*.

#### ii) Mean-Field Model of Clone Counts

Given a relationship such as Eq. 6 that determines *μ* ^∗^, we can explicitly develop a model that quantifies naive T cell subpopulations according to their immigration and proliferation rates *α* and *r*. For a given, realized value of *α* and *r*, we denote the expected number of clones of size *k* with these immigration and proliferation rates by $c_{k}\left(\alpha ,r\right)$ . The mean-field equations for the dynamics of these mean clone counts in the neutral model were derived in (39, 40) and are reviewed in Section 1 of the **Supplementary Material**. In a neutral model, we assume that all clones *Q* carry the same rates *α* and *r* so that the mean field evolution equation for $c_{k}\left(a,r\right)$ is given by solving (38, 39)


(7) $$\begin{array}{c} \frac{\text{d}c_{k}\left(\alpha ,r\right)}{\text{d}t}=\alpha \left[c_{k-1}\left(\alpha ,r\right)-c_{k}\left(\alpha ,r\right)\right]+r\left[\left(k-1\right)c_{k-1}\left(\alpha ,r\right)-kc_{k}\left(\alpha ,r\right)\right]\\ +\mu \left(N\right)[\left(k+1\right)c_{k+1}\left(\alpha ,r\right)-kc_{k}\left(\alpha ,r\right)], \end{array}$$

along with the constraint $\sum_{k=0}^{\infty }c_{k}\left(\alpha ,r\right)=c_{0}+\sum_{k=1}^{\infty }c_{k}\left(\alpha ,r\right)=Q$ . Note that $c_{k}\left(\alpha ,r\right)$ and *n_α,r_* are related via $\sum_{k=1}^{\infty }kc_{k}\left(\alpha ,r\right)=n_{\alpha ,r}$ . We use the notation *c_k_* to denote the predicted clone counts derived from our mathematical model to distinguish them from *measured* clone counts ${\hat{c}}_{k}$ . Equation 7 assumes that both $c_{k}\left(\alpha ,r\right)$ and *N* are uncorrelated, allowing us to write the last term as a product of functions of the mean population $N=\sum_{k=1}^{\infty }kc_{k}$ and $c_{k+1},c_{k}$ . Under steady-state, we approximate *μ*(*N*) by *μ* ^∗^ found by solving Eq. 6 as a function of $\lambda ,\bar{\alpha }$ , and the hyperparameters defining *π_r_* (*r*). The steady-state solution of Eq. 7 follows a negative binomial distribution with parameters α/*r* and *r*/*μ* ^∗^ < 1 (10, 39)


(8) $$c_{k\geq 1}\left(\alpha ,r,\mu^{*}\right)=Q{\left(1-\frac{r}{\mu^{*}}\right)}^{\alpha /r}{\left(\frac{r}{\mu^{*}}\right)}^{k}\frac{1}{k!}\prod_{\ell =0}^{k-1}\left(\frac{\alpha }{r}+\ell \right),$$

The predicted number of absent clones is $c_{0}=Q-\sum_{k=1}^{\infty }c_{k}\left(\alpha ,r,\mu^{*}\right)$ . The solution 8 depends implicitly on the parameter $\lambda /\bar{\alpha }$ through *μ* ^∗^ determined by Eq. 6. Although *c_k_* (*α*, *r*, *μ* ^∗^) has not yet been averaged over *α, r*, it also implicitly depends on *λ* and the parameters that define *π_r_* (*r*) through *μ* ^∗^ and Eq. 6. Specifically, larger *λ* leading to smaller *μ* ^∗^ results in a more slowly decaying *c_k_* (*α, r, μ* ^∗^) as a function of *k*. This behavior will be propagated through subsampling and averaging over *α* and *r*.

### Subsampling

Unless an animal is sacked and its entire naive T cell population is sequenced, TCR clone distributions are typically measured from sequencing TCRs in a small blood sample. In such samples, low population clones may be missed. In order to compare our predictions with measured clone abundance distributions, we must revise our predictions to allow for random cell sampling. We define *η* as the fraction of naive T cells in an organism that is drawn in a sample and assume that all naive T cells in the organism have the same probability *η* of being sampled. This is true only if naive T cells carrying different TCRs are not preferentially partitioned into different tissues and are uniformly distributed within an animal. Let us assume that a specific clone is represented by ℓ cells in an organism. If $N^{*}\eta \gg \ell$ , the probability that *k* cells are randomly sampled from the same clone approximately follows a binomial distribution with parameters ℓ and *η* (40–44)


(9) $$\mathbb{P}\left[k|\ell \right]\approx \left({}_{k}^{\ell }\right)\eta^{k}{\left(1-\eta \right)}^{\ell -k},\;k\leq \ell .$$

The associated mean *sampled* clone count $c_{k}^{s}$ depends on the predicted whole-organism clone count and ℙ[*k*|*ℓ*] via the formula


(10) $$\begin{array}{c} c_{k}^{s}\left(\alpha ,r,\mu^{*},\eta \right)\approx \sum_{\ell \geq k}c_{\ell }\left(\alpha ,r,\mu^{*}\right)\mathbb{P}\left[k|\ell \right]\\ =\sum_{\ell \geq k}c_{\ell }\left(\alpha ,r,\mu^{*}\right)\left({}_{k}^{\ell }\right)\eta^{k}{\left(1-n\right)}^{\ell -k}. \end{array}$$

where *c_ℓ_* (α, *r*, *μ* ^∗^) is determined by Eq. 8. Explicitly performing the sum in Eq. 10 yields the sampled clone count


(11) $$c_{k}^{s}\left(\alpha ,r,\mu^{*},\eta \right)=\frac{Q}{k!}{\left(\frac{\eta r/\mu^{*}}{1-\left(1-\eta \right)\left(r/\mu^{*}\right)}\right)}^{k}{\left(\frac{1-r/\mu^{*}}{1-\left(1-\eta \right)\left(r/\mu^{*}\right)}\right)}^{\frac{\alpha }{r}}\prod_{j=0}^{k-1}\left(\frac{\alpha }{r}+j\right).$$

The total expected number of clones in the sample (the richness) can be found via direct summation:


(12) $$C^{s}\left(\alpha ,r,\mu^{*},\eta \right)=\sum_{k=1}^{\infty }c_{k}^{s}\left(\alpha ,r,\mu^{*},\eta \right)=Q\left[1-{\left(\frac{1-r/\mu^{*}}{1-\left(1-\eta \right)r/\mu^{*}}\right)}^{\alpha /r}\right].$$

**Figure 3:**
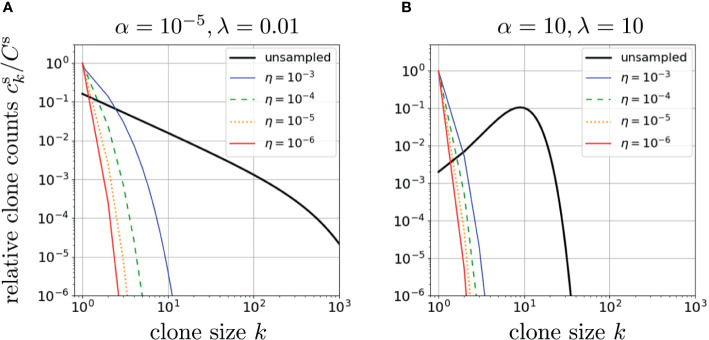
The effects of sampling on two different neutral-model relative clone counts $c_{k}^{s}/C^{s}$ plotted using the dimensionless proliferation rate *r* = 1/2 in Eqs. 11 and 12 or Eq. 10 and S9 from Section 2 of the **Supplementary Material**. In **(A)**, we used *α* = 10^-5^, *λ* =0.01. The effect of sampling is illustrated for *η* = 1 (no subsampling), 10^-3^, 10^-4^, 10^-5^, and 10^-6^. All clone counts are qualitatively similar, with subsampling increasing the exponential decay in *c_k_*. In **(B)**, we use a physiologically unrealistic set of parameters, *α* = *λ* = 10, which leads to a qualitatively different unsampled clone count pattern that exhibits a peak. However, under small subsampling fractions *η*, the clone count loses its peak as it shifts to a rapidly decreasing patterns $c_{k}^{s}$ that are not significantly different from sampled clone counts predicted using the parameters *α* and *λ* in **(A)**. This indicates inferring parameters using clone counts is ill-conditioned (rather insensitive to parameters) if *η* is too small.

As shown in **Figure 3**, random subsampling greatly affects the observed clone counts, with small sampling fractions *η* leading to fast decay in *k* of $c_{k}^{s}\left(\alpha ,r,\mu^{*},\eta \right)$ and shifting *c_k_* at large *k* to much smaller values of *k* while reducing the values of *c_k_* for small *k* (42). Note that setting *η* = 1 in Eq. 11 leads to Eq. 8, the whole-body clone count. In **Figures 3A, B** we plot results from our model using two very different dimensionless parameter sets, *α* = 10^-5^, *r* = 1/2, *λ* = 0.01, and *α = λ* =10, *r* = 1/2, to generate two qualitatively different patterns of neutral model clone counts *c_k_*. If the subsampling *η* ≪ 1 is sufficiently small, the resulting $c_{k}^{s}$ corresponding to the two qualitatively different *c_k_* can appear similar. This implies that small sampling fractions make the estimation of whole-body clone counts from sampled data somewhat ill-conditioned, *i.e.*, different whole-body clone counts, upon sampling, may yield similar sampled clone counts. Although sampling can strongly affect the inference of *c_k_*, immigration and proliferation rate distributions may also affect the observed clone count as we investigate below.

### Heterogeneity and Determination of *π* (*α*, *r | Ɵ*_0_)

The fundamental result given in Eq. 11 applies only to the clone count density in a neutral model in which the immigration and proliferation rates are *α* and *r* for all clones. We now average the sampled clone counts $c_{k}^{s}\left(\alpha ,r,\mu^{*},\eta \right)$ (Eq. 11) and the richness *C^s^* (*α*, *r*, *μ* ^∗^, *η*) (Eq. 12) over a distribution of immigration and proliferation rates *π*(*α, r*) to capture the heterogeneity across TCR clones. This final result can then be compared with experimentally measured clone counts. Recall that *π*(*α, r*) can depend on hyperparameters *θ*_0_ that define the shape of *π*. We then explicitly denote the distribution by *π*(*α*, *r|θ*_0_).

Once *π*(*α*, *r|θ*_0_) is defined, we can weight sampled clone counts accordingly. For example, one may assume $\theta_{0}=\left\{\bar{\alpha },w\right\}$ , with each of the two hyperparameters defining $\pi \left(\alpha ,r|\theta_{0}\right)=\pi_{\alpha }\left(\alpha |\bar{\alpha }\right)\pi_{r}\left(r|w\right)$ , leading to


$$c_{k}^{s}\left(\mu^{*},\eta ,\theta_{0}=\left\{\bar{\alpha },w\right\}\right)=\int_{0}^{\infty }\text{d}\alpha \int_{0}^{1}\text{d}r\pi \left(\alpha ,r|\theta_{0}\right)c_{k}^{s}\left(\alpha ,r,\mu^{*},\eta \right).$$

#### i) Proliferation Rate Heterogeneity

First, we consider a distribution of TCR sequence-dependent proliferation rates. Since TCR-antigen affinity depends on the receptor amino-acid sequence, the rate of T cell activation and subsequent proliferation can be clone-specific (31, 45). Thus, the specific interactions between TCRs and low-affinity MHC/self-peptide complexes maps to a distribution of proliferation rates among all the *Q* possible clones. Since there are no data (known to us) that can be used to infer this mapping or the specific shape of *π_r_* (*r*|*w*), we assume, for simplicity, a simple uniform “box” distribution centered about a mean value $\bar{r}=1/2$ :


(13) $$\pi_{r}\left(r|w\right)=\{{}_{0\;\text{otherwise}}^{1/w\;\text{if}\left|r-1/2\right|<w/2}$$

where 0 ≤ *w* ≤ 1 represents the relative width of the uniform box distribution. The minimum and maximum dimensionless proliferation rates in this distribution are then 1/2-*w*/2 and 1/2+*w*/2, respectively. The dimensionless self-consistency condition (Eq. 6) thus yields


(14) $$\mu^{*}=\frac{\left(\frac{1}{2}+\frac{w}{2}\right)e^{\lambda w/\bar{\alpha }}-\left(\frac{1}{2}-\frac{w}{2}\right)}{e^{\lambda w/\bar{\alpha }}-1}$$

**Figure 4:**
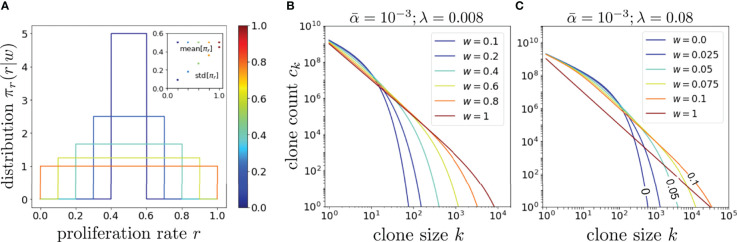
An exploration of the effects of proliferation rate heterogeneity on the mean clone counts *c_k_* with Q = 10^13^. **(A)** Various box distributions *π_r_* (*r*|*w*) for *w* = 0, 0.2, 0.4, 0.6, 0.8, and 1. **(B)** Using Eq. 14 and the dimensionless values $\bar{\alpha }=10^{-3},\lambda =8\times 10^{-3}$ such that $\lambda /\bar{\alpha }=8$ , we plot, using the same color spectrum as **(A)**, the corresponding clone counts *C_k_* and show that wider distributions typically generate longer-tailed *c_k_*. However, if *λ* is set even larger such that $\lambda /\bar{\alpha }=80$ even modest values of *w* can generate a very long-tailed *c_k_*, as shown in **(C)**. The color spectrum in **(C)** is for visualization only and not associated with that in **(A, B)**. In the limit of very large $\lambda /\bar{\alpha }$ , the effects of heterogeneous proliferation saturate at very small *w* beyond which it has negligible effect in further extending the tail.

To understand the effects of proliferation rate heterogeneity we begin by considering it effects on whole-organism (*η* = 1) clone counts. Since the function *c_k_* (*α*, *r*, *μ* ^∗^) defined by Eq. 8 contains the exponentially decaying term (*r*/*μ* ^∗^)*^k^*, a fixed dimensionless value of *μ* ^∗^ and *r* = 1/2 leads to an exponential decay in *c_k_* in *k*. However, if *w* > 0, different values of *r* and *μ* ^∗^ contribute to this decay term, yielding nontrivial behavior and a much slower decay as seen in **Figure 4** for $\lambda /\bar{\alpha }=8,80$ and different values of *w*.

#### ii) Immigration Rate Heterogeneity

Next, we use previous studies that predict V(D)J recombination frequencies associated with each TCR sequence to construct a distribution *π*_α_(α) for the TCR sequence-dependent thymic output. A statistical model for differential V(D)J recombination in humans is implemented in the Optimized Likelihood estimate of immunoGlobulin Amino-acid sequences (OLGA) software (28), which is an updated version of the Inference and Generation of Repertoires (IGoR) software (20). Below, we estimate $\pi_{\alpha }\left(\alpha |\bar{\alpha }\right)$ by sampling a large number of TCRs from OLGA that draws sequences according to their generation probability. Our working assumption is that thymic selection is uncorrelated with V(D)J recombination so the relative probabilities of forming different TCRs provide an accurate representation of the ratios of the TCRs exported into the periphery.

Both IGoR and OLGA can be used to generate the probabilities corresponding to each drawn sequence but this requires significant computational time and memory. Equivalently, since the sequence draws are proportional to the underlying probabilities, we simply drew N_*_ sequences and counted the frequencies of each amino acid sequence. Out of N_*_ sequence draws from IGoR or OLGA, there will be C_*_ distinct amino sequences (the richness of the drawn sequences). Since some sequences are drawn *j*>1 times, C_*_ ≤ N_*_. If *b_j_* distinct sequences are drawn *j* times, and the maximum observed frequency max{*j*} ≡ *J*, $C_{*}=\sum_{j=1}^{J}b_{j}$ , $N_{*}=\sum_{j=1}^{J}jb_{j}$ , while *b_j_*/C_*_ is the fraction of all drawn sequences that appear *j* times. For N_*_ = 10^9^, we found C_*_ = 372,806,648 ≈ 3.72 × 10^8^ and a maximum observed frequency max{*j*} = *J* = 52,294 for the alpha chain and C_*_ = 875,920,705 ≈ 8.76 × 10^8^ and *J* = 6430 for the beta chain.

We model the effective immigration rate of a TCR sequence drawn *j* times to be proportional to *j* so that *α_j_* ≡ α_*_j. To fix the proportionality α_*_, we identify the mean immigration rate averaged across the C_*_ observed sequences with the mean physiological rate $\bar{\alpha }$


(15) $$\alpha_{*}\sum_{j}\frac{jb_{j}}{C_{*}}\approx \bar{\alpha }$$

to find $\alpha_{*}=\bar{\alpha }C_{*}/N_{*}$ and thus


(16) $$\alpha_{j}=\frac{\bar{\alpha }j}{\left(N_{*}/C_{*}\right)}.$$

**Figure 5:**
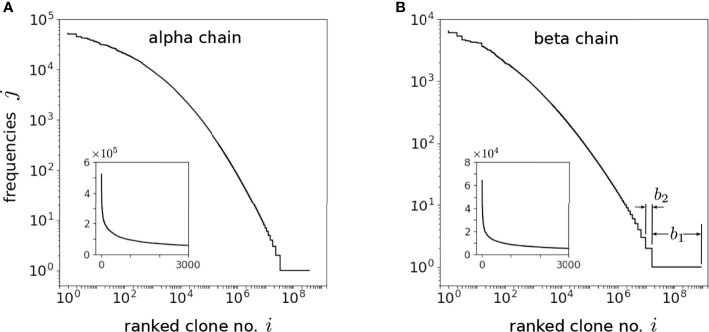
Ordered integer-valued frequencies *j*, plotted on a log-log scale, of the C_*_ distinct **(A)** alpha and **(B)** beta chains drawn using OLGA. The index 1 ≤ *i* ≤ C_*_ < N_*_ labels the distinct sequences drawn while *b_j_* is defined as the number these sequences that exhibit the specific frequency *j* [*b*_1_ and *b*_2_ are explicitly indicated in **(B)**]. The highest frequency clone appears *J* times such that *b_j>J_* = 0. Since C_*_ is comparable to N_*_, the drawn sequences are dominated by the low probability ones that appear only once. The insets display the frequencies on a linear scale and indicate the long-tailed behavior of the frequencies. The shape of the frequency spectra is self-similar once N_*_ ≳ 10^7^, allowing us to use this sampling procedure to reliably estimate $\pi_{\alpha }\left(\alpha |\bar{\alpha }\right)$ .

The frequencies *j* of the drawn realization of clones are plotted in decreasing order against the C_*_ distinct sequences in **Figures 5A, B**. From these frequencies *j* and the number of sequences *b_j_* exhibiting them, we approximate averages of any function *y*(*α*) over $\pi_{\alpha }\left(\alpha |\bar{\alpha }\right)$ by taking a sum over the values *α_j_*:


(17) $$\int \pi_{\alpha }\left(\alpha |\bar{\alpha }\right)y\left(\alpha \right)\approx \sum_{j=1}^{J}\frac{b_{j}}{C_{*}}y\left(\alpha_{j}\right).$$

Alternatively, when drawing sequences IGoR and OLGA (using the Pgen feature) one can also directly output their probabilities *p_i_*, whose values would be proportional to the frequency *j* if large numbers of sequences are drawn as described above. We can use these countable sequences and probabilities to construct *α* and *π_α_* (*α*) by defining $\alpha_{i}=\bar{\alpha }QC_{*}p_{i}/p_{T}$ where $p_{T}=\sum_{i=1}^{C_{*}}p_{i}$ . By plotting the values of *p_i_*, we arrive at a distribution similar to that shown in **Figure 5**. In this case too, we find that a large number of low-probability sequences dominates the averaging of clone counts using the distribution of immigration rates constructed using IGoR/OLGA.

Now that we have specified the distributions for $\pi_{\alpha }\left(\alpha |\bar{\alpha }\right)$ and *π_r_* (*r*|*w*), we can compute the mean, sampled, immigration- and proliferation-averaged clone counts and compare them with measurements. The full formula for the immigration and proliferation rate-averaged clone counts under subsampling is


(18) $$\begin{array}{c} c_{k}\left(\bar{\alpha },\mu^{*},w,\eta \right)=\int_{0}^{\infty }\text{d}\alpha \int_{0}^{1}\text{d}r\pi_{\alpha }(\alpha |\bar{\alpha })\pi_{r}\left(r|w\right)c_{k}^{s}\left(\alpha ,r,\mu^{*},\eta \right)\\ =\frac{Q}{k!}\sum_{j=1}^{J}\frac{b_{j}}{C_{*}}\int_{\left(1-w\right)/2}^{\left(1+w\right)/2}\frac{\text{d}r}{w}{\left(\frac{\eta r/\mu^{*}}{1-\left(1-\eta \right)r/\mu^{*}}\right)}^{k}\times {\left(\frac{1-r/\mu^{*}}{1-\left(1-\eta \right)r/\mu^{*}}\right)}^{\frac{\alpha_{j}}{r}}\prod_{i=0}^{k=1}(\frac{\alpha_{i}}{r}+i), \end{array}$$

where *α_j_* is given by Eq. 16 and *μ* ^∗^ is given by Eq. 14. Eq. 18 is our “full model” from which we make predictions of clones count-related quantities and compare them with data. Using this expression, we can mathematically study the importance of the heterogeneities in *α* and *r* by comparing predictions from simple forms of $\pi_{\alpha }\left(\alpha |\bar{\alpha }\right)$ and *π_r_* (*r*|*w*), as presented in Section 2 of the **Supplementary Material** to those derived from $\pi \left(\alpha ,r\right)=\delta \left(\alpha -\bar{\alpha }\right)\delta \left(r-\frac{1}{2}\right)$ of the neutral model.

From **Figure 5**, observe that $b_{1}\gg b_{j>1}$ . In fact, a majority of the naive T cell population is comprised of clones that are produced only once. The linear-scale insets also show a long tail indicating a large number of clones that are generated few times. Thus, for sufficiently small $\bar{\alpha }$ , our formulae for *c_k_* and all subsequent quantities can be approximated by taking the $\bar{\alpha }/r\ll 1$ limit. As we show in Section 3 of the **Supplementary Material**, such a simpler expression remains highly accurate, provided the dimensionless $\bar{\alpha }<10^{-2}$ , and allows efficient computation. This implies that the full result arising from averaging $c_{k}^{s}\left(\alpha ,r,\mu^{*},\eta \right)$ over $\pi_{\alpha }\left(\alpha |\bar{\alpha }\right)$ can also be approximated by using a single effective value $c_{k}^{s}\left(\bar{\alpha },r,\mu^{*},\eta \right)$ , supporting our overall conclusion that predicted heterogeneity in human T cell immigration rates do not appreciably influence clone count distributions. While physiological distributions $\pi_{\alpha }\left(\alpha |\bar{\alpha }\right)$ do not yield clone counts appreciably different from those of a neutral immigration model, small changes in proliferation rate heterogeneity *w* can significantly affect the clone count structure $c_{k}^{s}$ . Nonetheless, for completeness, we will perform the full summation over *α_j_* (Eq. 18). All parameters, hyperparameters, and variables used in our modeling and data analysis are listed in **Tables 1**, **2**.

**Table 2:** Model variables and their definitions.

variables	definition
*Q ∈ ℤ* ^+^	theoretical number of possible TCRs ~10^13^ – 10^18^ (36)
$\hat{N}\in {\mathbb{Z}}^{+}$	number of naive T cells in organism ~10^10^ – 10^11^ (5)
*N*(*t*) *∈* ℝ^+^	number of naive T cells from model
*N* ^∗^ *∈* ℝ^+^	steady-state number of naive T cells from model
*N^s^* ≡ *ηN* ^∗^ *∈* ℝ^+ ^	subsampled number of naive cells from model
N_*_ ∈ ℤ^+^	number of draws from IGoR/OLGA
$\hat{C}\in {\mathbb{Z}}^{+}$	total number of clones in organism (richness) ~10^6^ – 10^8^ (36)
${\hat{C}}^{s}\in {\mathbb{Z}}^{+}$	total number of sampled clones (sampled richness)
*C*(*θ*) *∈* ℝ^+^	total number of clones in organism from model
*C^s^* (*θ*,*η*) *∈* ℝ^+^	total number of sampled clones from model
C_*_ ∈ ℤ^+^	number of different sequences drawn from IGoR/OLGA
${\hat{c}}_{k}\in {\mathbb{Z}}^{+}$	discrete number of clones of size *k*
*c_k_* (*θ*) *∈* ℝ^+^	model of number of clones containing *k* cells
${\hat{c}}_{k}^{s}\in {\mathbb{Z}}^{+}$	discrete number clones of size *k* in sample
$c_{k}^{s}\left(\theta ,\eta \right)\in {\mathbb{R}}^{+}$	modeled number of sampled clones containing *k* cells
$f_{k}^{s}=\frac{k{\hat{c}}_{k}^{s}}{{\hat{C}}^{s}}\in [0,1]$	fraction of all sampled cells in clones of size *k*
$f_{k}^{s}\left(\theta ,\eta \right)=\frac{kc_{k}^{s}\left(\theta ,\eta \right)}{C^{s}\left(\theta ,\eta \right)}\in [0,1]$	modeled fraction of all sampled cells in clones of size *k*

The variables with $\hat{.}$ denote measured numbers, while populations written as functions of parameters θ are those predicted from our model (the dimensionless parameters used in our model are θ = {α,r}). The probability distributions *π*(α,r|θ_0_) are defined by hyperparameters θ_0_ (the dimensionless hyperparameters used in this study are $\theta_{0}=\left\{\bar{\alpha },w\right\}$ ). Upon averaging predicted quantities such as $c_{k}^{s}\left(\alpha ,r\right)$ over π(α, r|θ_0_) we find $c_{k}^{s}\left(\theta_{0}\right)$ .

## Results and Analysis

Before performing a quantitative comparison with measured clone counts from Oakes et al. (12), we discuss the qualitative features of our model and typical physiological parameter ranges. While even the basic model parameters are difficult to measure, our nondimensionalized model unifies the mechanisms and concepts common to the maintenance of diversity in the T cell repertoire across different organisms.

When considering the data, we observe that even after significant subsampling, there are appreciable clone counts at reasonably large clone sizes *k*, whereas the unsampled clone counts decay exponentially in *k* with rate log(*μ* ^∗^/*r*). Even though *r* may take on a range of values, as determined by π*_r_* (*r*), the slowest decay of *c_k_* arises from the largest possible values of *r*. Thus, a larger proliferation rate heterogeneity *w* will generally yield a longer-tailed *c_k_*, as illustrated in **Figure 4**. Since the data we analyze are derived from human samples, we will use the following arguments as a rough guide to the relevant range of parameters:

- The average total number of naive T cells is not completely known but is estimated to be about *N* ^∗^~ 10^11^ (35). However, the circulating population in the peripheral blood is approximately two orders of magnitude smaller. These circulating naive T cells nonetheless exchange with those in the much larger population in the lymph and other tissues. The timescale of this exchange (relative to the age of the organism being sampled or the intersample times) will determine the effective statistically accessible *N* ^∗^ relevant for sampling clone counts $c_{k}^{s}$ . We will use an order-of-magnitude estimate on the lower range of measurements and estimate *N* ^∗^~10^10^−10^11^.
- The theoretical total possible number *Q* of TCRs of either alpha or beta chains may be in the range 10^13^−10^18^ (46), but the actual number of clones with immigration rate *α_i_* that allows it to be produced even once in a lifetime is more relevant and probably much smaller. Thus, the effective value of *Q* may reside at the lower range, leading to λ ≡ *N* ^∗^/*Q* ~ 10^-4^−10^-2^.
- The average (dimensional) immigration rate per clone $\bar{\alpha }$ can be deduced from the total thymic output of all clones $\bar{\alpha }Q$ , which has been estimated across a wide range of values $\bar{\alpha }Q∼10^{7}-10^{8}$ /day (29, 47–50). If we use an effective repertoire size of *Q* ~ 10^13^−10^14^, the average per clone immigration rate becomes $\bar{\alpha }∼10^{-7}-10^{-5}$ /day.
- The mean proliferation rate *r* is difficult to measure but has been estimated to be on the order of $\bar{r}∼10^{-4}-10^{-3}$ /day (29). If we nondimensionalize using $R=2\bar{r}$ , the *dimensionless* $\bar{\alpha }∼10^{-4}-10^{-1}$ .
- The sampling fraction *η*, although in principle determined experimentally, is also hard to quantify due to the uncertainty in *N* ^∗^. Blood sampling volume fractions from humans are typically *η* ~ 10^-3^; however, in recent experiments (12) the number of enumerated sequences ~10^5^, which, given rough estimates of effective *N* ^∗^ ~ 10^10^-10^11^, yield *η* ~ 10^-6^ - 10^-4^. Due to this uncertainty in *η*, we will explore different fixed values of *η* around 10^-5^.

Using the above guide for reasonable parameter ranges, we now consider fitting our results in Eqs. 18, S9-S14 to some of the available data (12). Before doing so, note that although the log-log plots shown in **Figures 1A, B** provide a simple visual for $\log c_{k}^{s}$ or $\log\left[c_{k}^{s}/C^{s}\right]$ , fitting must be performed on the linear scale. The measured data includes data at values of *k* for which no clones were detected so that $c_{k}^{s}=0$ . These data points nonetheless should be included in the fitting as they represent realizations of the system. However, on the log scale these zero data points translate to $\log c_{k}^{s}\to -\infty$ so numerical fitting on the log-log scale could be misleading once a value of $c_{k}^{s}=0$ is encountered. Thus, we will fit our mean-field model on the linear scale to the *fraction* $f_{k}^{s}$ of the total number of sampled cells that are in clones of size *k*


(19) $$\begin{array}{c} f_{k}^{s}\left(\bar{\alpha },\lambda ,w,\eta \right)\equiv \frac{kc_{k}^{s}\left(\bar{\alpha },\lambda ,w,\eta \right)}{N^{s}}=\frac{kc_{k}^{s}\left(\bar{\alpha },\lambda ,w,\eta \right)}{\sum_{\ell =1}^{\infty }\ell c_{\ell }^{s}\left(\bar{\alpha },\lambda ,w,\eta \right)}\\ =\frac{kc_{k}^{s}\left(\bar{\alpha },\lambda ,w,\eta \right)}{Q\eta \lambda } \end{array}$$

where the denominator *Qηλ* comes directly from the definition $\sum_{\ell =1}^{\infty }\ell c_{\ell }^{s}(\bar{\alpha },\lambda |\eta )\equiv N^{s}$ , the sampling relation *N^s^* = *ηN* ^∗^, and Eq. 6. Note that we have switched the dependence from *μ* ^∗^ to *λ* (see Eq. 14). Rather than using *N^s^* directly from the number of reads in an experimental sample, equivalently, we use the model expression *N^s^* = *Qηλ* to arrive at the last equality in Eq. 19. This form ensures strict normalization and is independent of the unknown repertoire size *Q* since $c_{k}^{s}$ is proportional to *Q*. The implicit factor of *Q* in $c_{k}^{s}$ from Eq. 11 cancels the explicit *Q* in the denominator of Eq. 19 so that $f_{k}^{s}$ as well as $c_{k}^{s}/C^{s}$ depend on *Q* only through the determination of *μ* ^∗^ through *λ ≡ N* ^∗^/*Q* in Eq. 6.

Our mathematical framework provides only *mean* sampled clone counts while each sample of the data represents one realization. Large sample-to-sample variations in the clone counts would render the fitting less informative, but these large variations were not seen in the triplicate samples in Oakes et al. (12). Mechanistically, we expect that for large *k* the number of cells contributing to $f_{k}^{s}$ is also large so demographic stochasticity is relatively small and results in small uncertainties in the value of *k*, and not in the magnitude of $f_{k}^{s}$ . Large clones are also likely to include memory T cells that have been produced after antigen stimulation of specific clones. Memory T cells are difficult to accurately distinguish from naive T cells (12) but we will see that large *k* components of $f_{k}^{s}$ negligibly influence the fitting. We can now compare our model $f_{k}^{s}\left(\bar{\alpha },\lambda ,w,\eta \right)$ with the data $f_{k}^{s}$ (data) by constructing the error


(20) $$H\left(\bar{\alpha },\lambda ,w,\eta \right)=\sum_{k=1}^{\infty }|f_{k}^{s}\left(\mathrm{data}\right)-f_{k}^{s}\left(\bar{\alpha },\lambda ,w,\eta \right){|}^{2}$$

and exploring how it depends on the parameters $\bar{\alpha },\lambda ,w$ , and sampling fraction η. Our goal is to find relationships among the parameters $\lambda ,\bar{\alpha }$ , and *w* that minimize $H\left(\bar{\alpha },\lambda ,w,\eta \right)$ .

**Figure 6:**
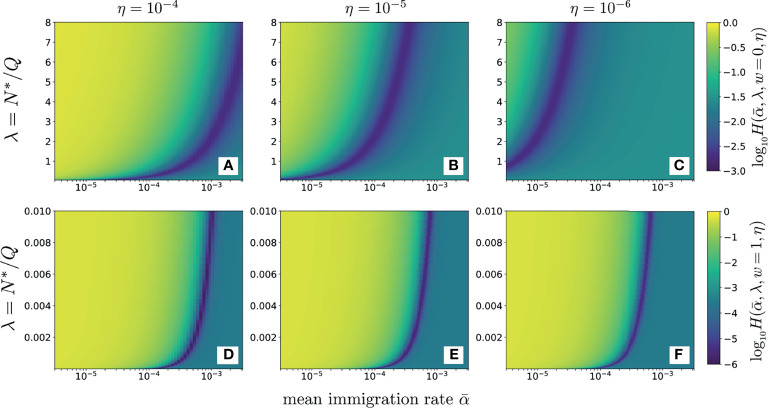
The error $H\left(\bar{\alpha },\lambda ,w,\eta \right)$ plotted as a function of *α* (on a log_10_ scale) and *λ*. Darker colors represent smaller values of error as shown by the scale bar on the right. The data used are the clone counts of beta chain sequences of naive CD4 cells from one patient, averaged over three samples. Panels **(A–C)** use the simple neutral model (Eqs. S9 and S10) and sampling fractions η = 10^-4^, 10^-5^, and 10^-6^, respectively. Since $\bar{\alpha }$ is on a log scale, the error is minimal along a line $\lambda_{\min}\propto \bar{\alpha }$ ; the error does not change appreciably along this path and only slightly decreases as *λ* and $\bar{\alpha }$ become smaller. For the neutral model (w = 0), the error is very sensitive to the sampling fraction *η*. Here, a fixed, physiologically reasonable value of $\bar{\alpha }$ results in a minimizing *λ*_min_ that is unreasonably large, in excess of one and that does not agree well with our expectations of $\lambda =N^{*}/Q\ll 1$ . Panels **(D–F)** show results for the distributed proliferation rate model at full width (*w* = 1). In this case, the errors are insensitive to the specific choice of η and the minimizing *λ*_min_ values are much smaller, consistent with our estimates of *N* ^∗^ and repertoire size. For *w* = 1, the values of the errors *H* are also smaller along the $\lambda_{min}-\bar{\alpha }$ minimum valley.

In **Figures 6A–C** the data $f_{k}^{s}$ (data) were derived from the average of three samples of beta chain CD4 sequences from one patient (12). These data, were used to compute and plot the error $H\left(\bar{\alpha },\lambda ,w=0,\eta \right)$ as a function of *λ* for various values of $\bar{\alpha }$ using the neutral model (*w* = 0, Eq. S9 in Section 2 of the **Supplementary Material**). For reasonable values of dimensionless $\bar{\alpha }\approx 10^{-5}-0.01$ and sampling fractions *η* = 10^-4^, 10^-5^, and 10^-6^, we find that the value of *λ* that minimizes $H\left(\bar{\alpha },\lambda ,w=0,\eta \right)$ , *λ*_min_, is typically 𝒪(1) or larger. In **Figures 6D–F** we use the full-width distribution *π_r_* (*r*|*w* = 1) to show the error for the same data using the same sampling fractions *η* = 10^-4^, 10^-5^, 10^-6^. Note that the values of *λ*_min_ are significantly smaller than those in found using *w* = 0 in **Figures 6A–C** and that the results are rather insensitive to the sampling fraction *η*. These smaller values of *λ*_min_ are more consistent with known physiological understanding. Thus, the distributed proliferation rate model provides a much more self-consistent fit to the data than the fixed proliferation rate neutral model. **Figure 6** also reveals that the values of *H* along the minimum valley are nearly constant, only slightly decreasing as $\bar{\alpha }\to 0$ . For each value of *α* we can identify the corresponding *λ*_min_ that minimizes *H*. However since the values of $H\left(\bar{\alpha },\lambda_{\min},w=0,\eta \right)$ for each $\left(\bar{\alpha },\lambda_{\min}\right)$ pair do not change appreciably, we cannot independently determine both.

**Figure 7:**
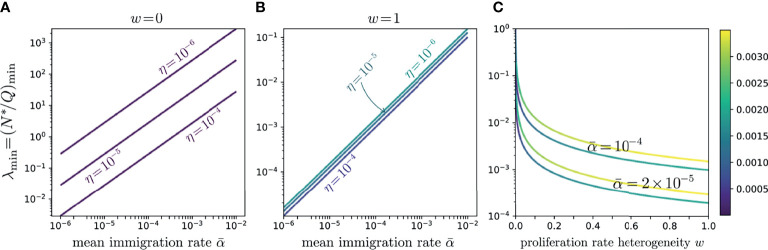
Log-log plots of *λ*_min_ values as functions of *α* for *η* = 10^-4^, 10^-5^, 10^-5^, and 10^-6^ for **(A)** the neutral model, *w* = 0, and **(B)** the full-width distributed proliferation rate model, *w* = 1. These curves trace the values of *λ*_min_ along the minimum valley in and show the relative insensitivity of the distributed proliferation rate model to the subsampling fraction *η*. In both **(A, B)**, the minimum line slopes are near one, with **(B)** showing a slightly greater slope, indicating *λ*_min_ is approximately proportional to $\bar{\alpha }$ over the entire range of *w*. The color intensity along the lines in **(A, B)** indicates variation in the total error along the minimum valley; their uniformity shows that the errors are nearly constant along each line. **(C)** Log-linear plot of *λ*_min_ as a function of proliferation rate heterogeneity *w* for $\bar{\alpha }=2\times 10^{-5},10^{-4}$ . The lower darker curves in each pair correspond to *η* = 10^-4^ while the lighter curves correspond to *η* = 10^-6^. The curves show that even a small heterogeneity *w* quickly reduces *λ*_min_ to below one; however, if *λ* is forced to be even smaller, the required heterogeneity *w* increases.

An alternate representation is shown in **Figure 7** where the relationship between $\bar{\alpha }$ and *λ*_min_ is seen to be approximately linear for both the neutral model (*w* = 0) and the heterogeneous, full-width model (*w* = 1). The color shading represents the corresponding value of $H\left(\bar{\alpha },\lambda_{\min},w,\eta \right)$ . One major observation is that the full-width case yields values of $\left(\bar{\alpha },\lambda_{\min}\right)$ that are closer to measured and expected physiological values and that these results are also less sensitive to *η* compared to those of the neutral case. On the other hand, although the variation in *H* is negligible across $\bar{\alpha }$ in both cases, the fully heterogeneous model (*w* = 1) carries a slightly larger error than the neutral one (w = 0). This is solely a consequence of our use of $f_{k}^{s}$ which weights the small *k* values significantly more in the fitting.

Since experimentally we expect small *λ*, we also investigate whether small errors *H* emerge for values of $\left(\bar{\alpha },\lambda_{\min}\ll 1\right)$ at intermediate 0 < *w* < 1. In **Figure 7C**, we plot *λ*_min_ as a function of *w* for various values of $\bar{\alpha }$ . Note that even small *w* significantly reduces, relative to the neutral case, the corresponding *λ*_min_. However, if our target is *λ*_min_ ∼ 10^-4^-10^-3^, the required *w* can become quite large. These results indicate that more heterogeneity is associated with more realistic values of the experimentally observed values of *N* ^∗^/*Q*.

**Figure 8:**
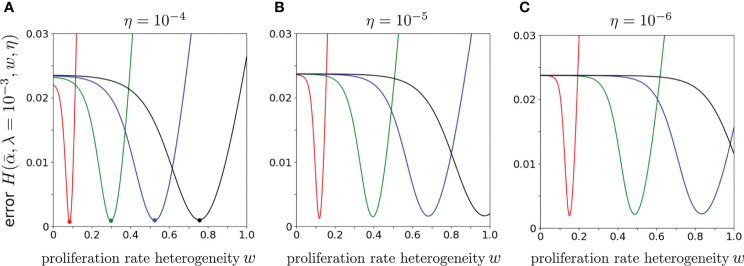
The error $H\left(\bar{\alpha },\lambda ,w,\eta \right)$ using CD4 alpha data from Oakes et al. (12) plotted as a function of *w* for various $\lambda /\bar{\alpha }$ . We fixed *λ* = 10^-3^ and varied, from left to right, $\bar{\alpha }=2\times 10^{-5}$ (red), 6 × 10^-5^ (green), 10^-4^ (blue) and 1.4 × 10^-4^ (black). From **(A–C)**, *η* = 10^-4^, 10^-5^, and 10^-6^. Smaller values of $\lambda /\bar{\alpha }$ result in larger best-fit values of *w*.

**Figure 9:**
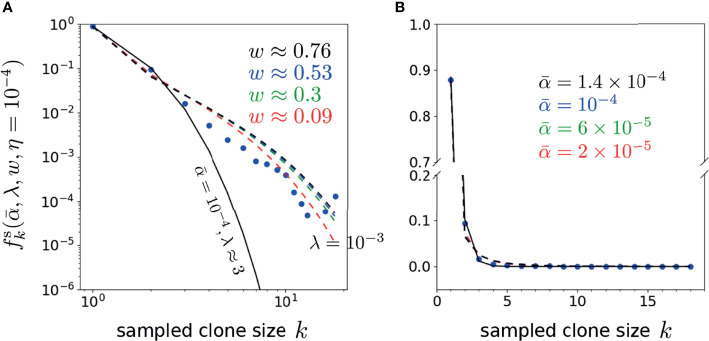
Plots of the representative optimal solutions of clone counts $f_{k}^{s}$ from Eq. 19 (using *η* = 10^-4^ and λ = 10^-3^ unless otherwise indicated) plotted along side the shown data from Oakes et al. (12). The model predictions and CD4 beta chain data are shown in both **(A)** log-log and **(B)** linear scales (there are no zero-values clone counts in this dataset). In **(A)**, the best fit model for the neutral model (*w* = 0 and $\pi_{\alpha }\left(\alpha |\bar{\alpha }\right)=\delta \left(\alpha -\bar{\alpha }\right)$ ) using $\bar{\alpha }=10^{-4}$ is given by λ ≈ 3 shown by the solid black curve. The dashed curves represents best-fit curves using the values associated with the error minima in, where $\bar{\alpha }=2\times 10^{-5}$ , *w* ≈ 0.09 (red), 6 × 10^-5^, *w* ≈ 0.3 (green), 10^-4^, *w* ≈ 0.53 (blue) and 1.4 × 10^-4^, *w* ≈ 0.76 (black). Note that the neutral model fits well for only the first 2-3 *k*-points, while the heterogeneous model (*w > 0*) fits better at larger *k*.

Finally, to explore the dependence of the error on the proliferation rate heterogeneity *w*, we fix $\bar{\alpha },\lambda$ , and *η*, and plot $H\left(\bar{\alpha },\lambda ,w,\eta \right)$ as a function of *w*. **Figure 8** shows that the *H*-minimizing *w* is very sensitive to $\lambda /\bar{\alpha }$ : for fixed *η*, as $\lambda /\bar{\alpha }$ is decreased the error is lowest for larger proliferation heterogeneity *w*. The minimum value of $H\left(\bar{\alpha },\lambda ,w,\eta \right)$ , however, is rather insensitive to $\lambda /\bar{\alpha }$ for all chosen *η*. Hence, near-optimal solutions with λ≪1 can be found when the proliferation rate heterogeneity *w* is appreciable. Using the parameters associated with the minima in **Figure 8A** (*η* = 10^-4^), we plot our predicted $f_{k}^{s}$ against the data $f_{k}^{s}$ (data) in **Figure 9**. As can be seen, when proliferation rate heterogeneity is allowed, the best-fits have small error and are found using realistic values, λ≪1 . Note that most of the information in the data lies in how $f_{k}^{s}$ (data) decreases over the first few values of *k*. The neutral model (*w* = 0) fits best for small values of *k*, but the corresponding values of *λ* and $\bar{\alpha }$ are too large and small, respectively. The goodness of fit of our model to the data depends mostly on the predicted initial decreases in $f_{k}^{s}\left(\bar{\alpha },\lambda ,w,\eta \right)$ . The constraints among the parameters $\lambda ,\bar{\alpha },w$ , and *η* derived from our model and can be applied to different clone counts such as the data shown in **Figure 1**. However, due to the ill-conditioning when *η* ≪ 1, the differences in these constraints across different data sets do not vary appreciably are only quantitatively different. Generally, the more rapidly decaying a clone count, the smaller the *w*, the smaller the *η*, of the larger the *λ*, all else being equal.

## Discussion

Here, we review and justify a number of critical biological assumptions and mathematical approximations used in our analysis. The effects of relaxing our approximations are also discussed.

### Distinct T Cell Components

It is known that naive T cells can change in time, with recent thymic emigrants evolving into mature naive T cells that carry different proliferation and death rates (51). For simplicity, we have assumed a single naive T cell compartment. To incorporate naive T cell evolution, we can allow the distribution *π_r_* (*r*) to evolve in time to reflect the relative abundances of T cell subpopulations, or, one can explicitly include multiple compartments, with cells from a recent emigrant compartment transitioning into a mature compartment. Each compartment would be described by its own steady-state death rates, clone counts, and distributions of proliferation rates. An analysis of a related sequential cell state transition model has been developed for clonal tracking in hematopoiesis (41).

### Factorization of π (α, r)

For mathematical tractability, we have assumed $\pi \left(\alpha ,r|\theta_{0}\right)=\pi_{\alpha }\left(\alpha |\bar{\alpha }\right)\pi_{r}\left(r|w\right)$ . Given the typical physiological values of $\bar{\alpha }$ , the clone count formulae derived from our model can be accurately approximated by a single value of $\bar{\alpha }$ . Thus, we expect that the immigration rate distribution can be approximated by $\pi_{\alpha }\left(\alpha |\bar{\alpha }\right)=\delta \left(\alpha -\bar{\alpha }\right)$ . This allows further approximation of our formulae as shown in Section 3 of the **Supplementary Material**. In Section 4 of the **Supplementary Material**, we explicitly show that factorisation is an accurate approximation.

We have also assumed that selection is uncorrelated with the generation probabilities of the TCR nucleotide sequences encoded in IGoR/OLGA. The assumption is that the recombination statistics are uncorrelated with the statistics of thymic selection, a process that is based on TCR amino acid sequences. However, we note that it has been suggested that selection pressure may induce a correlation between TCRs generated and selected (52). The corresponding statistics of the frequencies of *selected* TCRs would be modified from those of the *generated* TCRs shown in **Figure 5**. Nonetheless, we assume that the resulting distribution can still be approximated by a single-*α* model which will not qualitatively alter our conclusions.

### Mean-Field Approximation

Our mean-field approximation for the mean clone count *c_k_* is embodied in Eq. 7, where correlations between fluctuations in the total population $N=\sum_{k}kc_{k}$ in the regulation term *μ*(*N*) and the explicit *c_k_* terms are neglected. This approximation has been shown to be accurate for *k* ≲ *N* ^∗^ when $\bar{\alpha }Q^{2}>\mu \left(N^{*}\right)$ (39). The mean-field results overestimate the clone counts for *k* ≲ *N* ^∗^. Moreover, when the total steady-state T cell immigration rate is extremely small, the effects of competitive exclusion dominate and a single large clone arises (39, 53, 54). Nonetheless, an accurate approximation for the steady-state clone abundance *c_k_* can be obtained using a variation of the two-species Moran model as shown in (39). For the naive T cell system, because *Q* is so large, the mean immigration rate $\bar{\alpha }$ is such that competitive exclusion is not a dominant feature. Moreover, since *N* ^∗^ ≳ 10^10^, clones counts at comparable sizes are not observed and predicted to be negligible in all models. Since the values of $f_{k}^{s}$ (data) become exponentially smaller for large *k*, our inference is most sensitive to the values of $f_{k}^{s}$ (data) for small to modest *k*. The information in the data is primarily manifested by how the $f_{k}^{s}$ (data) decays in *k*, we before the mean-field approximation deviates from the exact solution. Thus, the parameters associated with the human adaptive immune system satisfy the conditions for the mean-field approximation to be accurate, justifying its use in the BDI model.

### Steady State Assumption

In this study, we only considered the steady state of our birth-death-immigration model in Eq. 8 because this limit allowed relatively easy derivations of analytical results. This was also the strategy for previous modeling work (4, 6, 7, 38, 39). However, the per-clone immigration and proliferation times may be on the order of months or years, a time scale over which thymic output diminishes as an individual ages (29, 55–57). Indeed, clone abundance distributions have been shown to show specific patterns as a function of age (58–60). Although *N*(*t*), with fixed $\bar{\alpha }$ and $\bar{r}$ relaxes to steady-state quickly, on a timescale of months, the different subpopulations of specific sizes described by their number *c_k_* relax to quasi-steady-state across a spectrum of time scales depending on the clone sizes *k* (39, 61). The timescales of relaxation of the largest clones can be estimated from the eigenvalues of the linearized system (Eqs. 7) and are found to be ~ 10 years. Thymic involution could be modeled by using a time-dependent *α*(*t*) that slowly decreases with age (57). Although T cells are thought to be primarily maintained through proliferation, thymic regeneration has also been shown to affect the naive T cell pool many years after thymectomy in infants. Here, a time dependent increase in *α*(*t*) after early thymectomy could be used. Indeed, the clone counts may be determined in early life (17) suggesting the dynamics of certain clones may be very slow, precluding a strict steady-state analysis for the entire repertoire.

In addition to time-dependent changes in *α*, more subtle time-inhomogeneities such as changes in proliferation and death rates have been demonstrated (55, 56, 62). Thus, our steady-state assumption could be relaxed by incorporation of time-dependent perturbations to the model parameters *μ* ^∗^ and/or π(*α, r*). Longitudinal measurements of clone abundances or experiments involving time-dependent perturbations would provide significant insight into the overall dynamics of clone abundances. The timescales required to reach steady state fall between 1/( $\bar{\alpha }Q$ ) and $1/\bar{\alpha }$ . Thus, it is possible that some components of *c_k_* does not reach steady state in an organism’s lifetime and our steady state model might not be be valid for all values of *c_k_* (57, 61) and a dynamic approach must be taken.

### Clustered Immigration

Our mean field model assumed that each immigration event introduced a single naive T cell in the immune system. However, T cells can divide before leaving the thymus and reach a homeostatic state in the periphery. This process can be described by the simultaneous immigration of more than one naive T cell with the same TCR. Clustered immigration of *q* cells can be implemented in the core model for *c_k_* (Eq. 7) via an immigration term of the form *α_q_* (*c_k-q_* (*α_q_*, *r*)-*c_k_* (*α_q_*, r)), where *c_k-q_* = 0 for *k-q < 0* (see Section 5 of the **Supplementary Material**). For *q* > 1, an informative analytic expression for *c_k_* is not available. In Figure S2 of the Section 5 of the **Supplementary Material**, we show the predicted clone abundance *c_k_* for a neutral model in which *q* = 5. When compared to the case where there is only one cell per immigration, the clone abundance *c_k_* will have a larger slope for *k* ⪅ *q*, making it kink more downward near *k ≈ q*. Thus, from **Figures S2** and **9A**, we can see that paired immigration (*q* = 2) would increase $f_{k}^{s}$ for *k* = 2, providing an improved fitting to data over single copy immigration (*q* = 1).

Thus, in addition to appreciable sensitivity of the predicted clone counts to π*_r_* (*r*|*w*), we also expect clustered immigration defined through the immigration rates α*_q_*, q > 1 to control the goodness of fit to data. Indeed, **Figure S2** suggests that the distribution of immigration cluster sizes *q*, in addition to the proliferation rate heterogeneity *w*, is an important determinant of measured clone counts and that *α_q_* may be constrained by data. We leave this for future investigation.

### General Conclusions

We developed a heterogeneous multispecies birth-death-immigration model and analyzed it in the context of T cell clonal heterogeneity; the clone abundance distribution is derived in the mean-field limit. Unlike previous studies (4), our modeling approach incorporated sampling statistics and provided simple formulae, allowing us to predict clone abundances under different rate distributions for arbitrarily large systems (*N* ^∗^ ∼ 10^10^ - 10^11^), without the need for simulation. The properties of the BDI model and the overall shape of the sampled clone count data renders the first few *k*-values of $c_{k}^{s}$ or $f_{k}^{s}$ the most important for determining the constraints among the model parameters. In other words, only the initial rate of the decrease in $f_{k}^{s}$ (data) for small *k* governs the quality of fitting to the model, and one should not expect to be able to explicitly infer more than one or two free parameters.

Our heterogeneous BDI model produced mean sampled clone count distributions that we could directly compare with measured clone counts. The unsampled clone counts *c_k_* of the neutral model (homogeneous *α* and *r*) follow a negative binomial distribution which is further modified upon sampling and distribution over the heterogeneous immigration and proliferation rates. Although we determined $\pi_{\alpha }\left(\alpha |\bar{\alpha }\right)$ through a code that implemented recombination statistics inferred from cDNA and gDNA sequences (20, 28), we found that the behavior of the model is rather insensitive to distributions $\pi_{\alpha }\left(\alpha |\bar{\alpha }\right)$ with mean values $\bar{\alpha }$ much smaller than the largest proliferation rates *r*. The model results are dominated by many low immigration-rate clones and a model that replaces *α* with its mean value $\bar{\alpha }$ is sufficient.

Conversely, we find that the shape of the clone count profiles *c_k_* are quite sensitive to the proliferation rate heterogeneity *w*. A small amount of heterogeneity quickly reduces the best-fit values of *λ* to reasonable values. For estimated values *η* ~ 10^-6^ – 10^-4^, $\bar{\alpha }∼10^{-4}$ , and small values of *λ* = *N* ^∗^/*Q* ≲ 10^-3^, requires a best-fit width *w* ≈ 1. Heterogeneity is needed to generate clones of sufficiently large size that persist after sampling. Although the number of TCR clones with large proliferation rates *r* may be small, such clones proliferate more rapidly contributing to higher clone counts at larger sizes. In particular, we found that the shape of expected clone abundance is sensitive to the behavior of the proliferation rate distribution near the maximum dimensional proliferation rate *R, π_r_* (*r* ≈ *R*). The predicted clone counts are also modestly sensitive to the distribution of immigration cluster sizes *q* (representing transient proliferation just before thymic output). When *q* > 1 cells of a clone are simultaneously exported by the thymus, the predicted mean clone counts decay much more slowly for small *k* ≲ *q* (see **Figure S2**). This modification will allow for better fitting since clustered immigration increases the predicted clone counts for larger $k,c_{2}^{s},c_{3}^{s}$ , etc., and eventually $f_{2}^{s}$ , $f_{3}^{s}$ , etc. Thus, we expect that a model containing multiple clustered immigration rates *α_q≥1_* will lower the error and provide better fitting, particularly at larger *w*. Additional analysis using a distribution of immigration cluster sizes may allow this type of clone count data to reveal more information about the physiological mechanism of naive T cell maintenance.

Even assuming modest heterogeneity, our work leads to the conclusion that the typical immigration heterogeneity is not enough to influence measured clone counts and that varying levels of proliferation heterogeneity is needed to shape $c_{k}^{s}$ (and $f_{k}^{s}$ ) (12). These results are consistent with the finding that naive T cells in humans are maintained by proliferation rather than thymic output (9). Since we have only investigated the effects of a uniform distribution for *π_r_* (*r*|*w*), further studies using more complex shapes of *π*(*α*, *r*|*θ*_0_) can be easily explored numerically using our modeling framework. Different parameter values and rate distributions appropriate for mice, in which naive T cells are maintained by thymic output, should also be explored within our modeling framework. Finally, it will be important to extend our steady-state model to allow *α*(t), *π_r_* (*r*,*t*), and *μ* ^∗^(*t*) to be functions of time in order to predict clone abundances in the presence of thymic involution and reduced proliferation with age (62, 63), which can even arise differentially in different compartments (64).

## Data Availability Statement

Publicly available datasets were analyzed in this study. This data can be found here: https://www.frontiersin.org/articles/10.3389/fimmu.2017.01267/full.

## Author Contributions

RD, TC, and MRD developed and analyzed the model and wrote the manuscript. YP organized published data, and DM assisted in sorting and organizing generated data. TC, YP, and MX performed numerical analyses and data fitting. All authors contributed to the article and approved the submitted version.

## Funding

This work was supported by grants from the NIH through grant R01HL146552 (TC), the Army Research Office through grant W911NF-18-1-0345 (MRD), the NSF through grants DMS-1814364 (TC) and DMS-1814090 (MRD). The authors also thank the Collaboratory in Institute for Quantitative and Computational Biosciences at UCLA for support to RD.

## Conflict of Interest

The authors declare that the research was conducted in the absence of any commercial or financial relationships that could be construed as a potential conflict of interest.

## Publisher’s Note

All claims expressed in this article are solely those of the authors and do not necessarily represent those of their affiliated organizations, or those of the publisher, the editors and the reviewers. Any product that may be evaluated in this article, or claim that may be made by its manufacturer, is not guaranteed or endorsed by the publisher.
