# Supplementary material for: How Naive T-Cell Clone Counts Are Shaped By Heterogeneous Thymic Output and Homeostatic Proliferation
Source: Front Immunol. 2022 Feb 17;12:735135. doi: 10.3389/fimmu.2021.735135 (PMC8891377; doi:10.3389/fimmu.2021.735135)
Supplement: Supplementary file 1 [file Presentation_1.pdf]

Supplementary Material

1 NEUTRAL MODEL

Here, we review the neutral BDI model in which there is no heterogeneity in either proliferation or immigration rates, $\pi(\alpha, r) = \delta(\alpha - \bar{\alpha})\delta(r - \bar{r})$. Upon inserting this expression for $\pi(\alpha, r)$ in Eq. 8, we find that the clone abundance c_k follows a negative binomial distribution (**?**):

$$c_k = Q\left(1 - \frac{\bar{r}}{\mu^*}\right)^{\bar{\alpha}/\bar{r}} \left(\frac{\bar{r}}{\mu^*}\right)^k \frac{1}{k!} \prod_{\ell=0}^{k-1} \left(\frac{\bar{\alpha}}{\bar{r}} + \ell\right). \tag{S1}$$

We can also express c_k/C, the clone abundance distribution normalized by the mean richness C in the body, as

$$\frac{c_k}{C} = \frac{c_k}{\sum_{\ell \geq 1} c_\ell} \tag{S2}$$

where $C = \sum_{\ell=1} c_\ell = Q(1 - (1 - \bar{r}/\mu^*)^{\bar{\alpha}/\bar{r}})$ is $C^{\rm s}$ in Eq. 12 with $\eta = 1$. Using $\bar{\alpha} \approx 1.6 \times 10^{-8}$/day, $\bar{r} \sim 5 \times 10^{-4}$/day, and $\mu^* \approx 6.4 \times 10^{-4}$, we find $\bar{\alpha}/\bar{r} \ll \bar{r}/\mu^* \sim O(1)$. The $\bar{\alpha}/\bar{r} \ll 1$ regime allows us to approximate c_k/C as a log-series distribution with parameter $\bar{r}/\mu^*$. To mathematically show this, consider a random variable X that follows a negative binomial distribution of parameters $\bar{\alpha}/\bar{r}$ and $\bar{r}/\mu^*$

$$\mathbb{P}\left[X = k\right] = \left(1 - \frac{\bar{r}}{\mu^*}\right)^{\bar{\alpha}/\bar{r}} \left(\frac{\bar{r}}{\mu^*}\right)^k \frac{1}{k!} \prod_{\ell=0}^{k-1} \left(\frac{\bar{\alpha}}{\bar{r}} + \ell\right). \tag{S3}$$

Note that the probability mass function of X above is also given by c_k/Q as can be seen from Eq. S1, the clone abundance distribution for all possible Q clones, which includes c_0, the number of all clones that are not represented in the organism. To find the clone abundance distribution c_k/C, for all the C clones present in the organism, we must exclude the case $k = 0$ by marginalizing the distribution of X over all $X > 0$:

$$\mathbb{P}\left[X = k | X > 0\right] = \frac{\mathbb{P}\left[X = k\right]}{\sum_{\ell \geq 1} \mathbb{P}\left[X = \ell\right]} = \frac{c_k/Q}{\sum_{\ell \geq 1} c_\ell/Q} = \frac{c_k}{C}. \tag{S4}$$

What remains is to show that the distribution in Eq. S4 converges to a log-series distribution of parameter $\bar{r}/\mu^*$ when $\bar{\alpha}/\bar{r} \to 0$. Consider the moment generating function of $X|X > 0$ given by

$$\mathbb{E}\left[e^{\xi X} | X > 0\right] = \frac{\mathbb{E}\left[e^{\xi X}\right] - \mathbb{E}\left[e^{\xi X} | X = 0\right] \mathbb{P}\left[X = 0\right]}{\mathbb{P}\left[X > 0\right]}. \tag{S5}$$

Since the moment generating function of a negative binomial distribution $\mathbb{E}\left[e^{\xi X}\right]$ is known, and since $\mathbb{P}\left[X > 0\right] = 1 - \mathbb{P}\left[X = 0\right]$ (see Eq. S3), we can write

$$\mathbb{E}\left[e^{\xi X}|X>0\right]=\frac{\left(\frac{1-\bar{r}/\mu^*}{1-e^{\xi}\bar{r}/\mu^*}\right)^{\bar{\alpha}/\bar{r}}-\left(1-\frac{\bar{r}}{\mu^*}\right)^{\bar{\alpha}/\bar{r}}}{1-\left(1-\frac{\bar{r}}{\mu^*}\right)^{\bar{\alpha}/\bar{r}}}. \tag{S6}$$

For any $x>0$, the limit $\bar{\alpha}/\bar{r}\to 0$ yields $x^{\bar{\alpha}/\bar{r}}=1+(\bar{\alpha}/\bar{r})\log x+o\left(\bar{\alpha}/\bar{r}\right)$. If we apply this result to Eq. S6 for $\mathbb{E}\left[e^{\xi X}|X>0\right]$, we find

$$\begin{aligned}\mathbb{E}\left[e^{\xi X}|X>0\right]&=\frac{1+\frac{\bar{\alpha}}{\bar{r}}\log\left(\frac{\mu^*-\bar{r}}{\mu^*-e^{\xi}\bar{r}}\right)-\left(1+\frac{\bar{\alpha}}{\bar{r}}\log\left(1-\frac{\bar{r}}{\mu^*}\right)\right)+o\left(\frac{\bar{\alpha}}{\bar{r}}\right)}{-\frac{\bar{\alpha}}{\bar{r}}\log\left(1-\frac{\bar{r}}{\mu^*}\right)+o\left(\frac{\bar{\alpha}}{\bar{r}}\right)}\\&=\frac{\log\left(1-e^{\xi}\frac{\bar{r}}{\mu^*}\right)}{\log\left(1-\frac{\bar{r}}{\mu^*}\right)}+o\left(1\right),\end{aligned}$$

which we recognize as the moment generating function of a log series distribution of parameter $\bar{r}/\mu^*$. Thus, we finally have

$$\lim_{\bar{\alpha}/\bar{r}\to 0}\frac{c_k}{C}=\frac{(\bar{r}/\mu^*)^k}{k\log\left(\frac{1}{1-\bar{r}/\mu^*}\right)}. \tag{S7}$$

2 EXPLICIT FORMS USING DIFFERENT IMMIGRATION AND PROLIFERATION RATE DISTRIBUTIONS

In the following, we propose four simplifying expressions for the heterogeneity-averaged clone counts $c_k^{\mathrm{s}}(\bar{\alpha},\mu^*,w,\eta)$ derived from Eq. 18.

Clone-independent Neutral model: $\pi(\alpha,r)=\delta(\alpha-\bar{\alpha})\delta(r-\bar{r})$

First, consider the simplest case where all naive T cells carry the same immigration and proliferation rates $\bar{\alpha}$ and $\bar{r}$, respectively, and define $\pi(\alpha,r)=\delta(\alpha-\bar{\alpha})\delta(r-\bar{r})$. This case corresponds to $w\to 0$ and $r\to\bar{r}=1/2$ in the $\pi_r(r|w)$ box distribution in Eq. 13. The self-consistent condition for μ^* and $\bar{\alpha}/\bar{r}$ become

$$\frac{\bar{r}}{\mu^*}\to\frac{\lambda}{\lambda+2\bar{\alpha}},\quad \frac{\bar{\alpha}}{\bar{r}}\to 2\bar{\alpha}, \tag{S8}$$

and the clone count given in Eq. 11 can be explicitly simplified to

$$c_k^{\mathrm{s}}(\bar{\alpha},\lambda,\eta)\equiv\frac{Q}{k!}\left(\frac{\eta\lambda}{\eta\lambda+2\bar{\alpha}}\right)^k\left(\frac{2\bar{\alpha}}{\eta\lambda+2\bar{\alpha}}\right)^{2\bar{\alpha}}\prod_{\ell=0}^{k-1}(2\bar{\alpha}+\ell). \tag{S9}$$

The total sampled clone count is then

$$C^{\mathrm{s}}(\bar{\alpha},\lambda,\eta)=\sum_{k=1}^{\infty}c_k^{\mathrm{s}}(\bar{\alpha},\lambda,\eta)=Q\left[1-\left(\frac{2\bar{\alpha}}{\eta\lambda+2\bar{\alpha}}\right)^{2\bar{\alpha}}\right]. \tag{S10}$$

Fixed immigration rate, distributed proliferation: $\pi(\alpha,r)=\delta(\alpha-\bar{\alpha})\pi_r(r)$

Next, consider a common immigration rate $\bar{\alpha}$ for all T cell clones and a box distribution $\pi_r(r|w)$ of full width $w=1$. Eq. 14 yields $\mu^*=(1-e^{-\lambda/\bar{\alpha}})^{-1}$, so that the averaged clone counts from Eq. 11 are now explicitly

$$c_k^{\mathrm{s}}(\bar{\alpha},\lambda,\eta)\equiv\frac{Q}{k!}\int_0^1\mathrm{d}r\left(\frac{\eta r/\mu^*}{1-(1-\eta)r/\mu^*}\right)^k\left(\frac{1-r/\mu^*}{1-(1-\eta)r/\mu^*}\right)^{\frac{\bar{\alpha}}{r}}\prod_{j=0}^{k-1}\left(\frac{\bar{\alpha}}{r}+j\right). \tag{S11}$$

The total sampled clone count can also be explicitly expressed as the integral over $C^{\mathrm{s}}(\bar{\alpha},r,\lambda|\eta)$ from Eq. 12:

$$C^{\mathrm{s}}(\bar{\alpha},\lambda,\eta)=Q\int_0^1\mathrm{d}r\left[1-\left(\frac{1-r/\mu^*}{1-(1-\eta)r/\mu^*}\right)^{\bar{\alpha}/r}\right]. \tag{S12}$$

Clone-specific immigration, fixed proliferation rate: $\pi(\alpha,r)=\pi_\alpha(\alpha|\bar{\alpha})\delta(r-\bar{r})$

Finally, we consider the case whereby all proliferation occurs at a fixed rate $\bar{r}$ and α is distributed according to Eq. 17, as determined from our OLGA sequence-drawing analysis. Using the same rate dimensionalization as before (Eqs. S8), we find explicitly

$$c_k^{\mathrm{s}}(\bar{\alpha},\lambda,\eta)=\frac{Q}{k!}\left(\frac{\eta\lambda}{\eta\lambda+2\bar{\alpha}}\right)^k\sum_{j=1}^{J}\frac{b_j}{C_\star}\left(\frac{2\bar{\alpha}}{\eta\lambda+2\bar{\alpha}}\right)^{2\alpha_j}\prod_{\ell=0}^{k-1}(2\alpha_j+\ell), \tag{S13}$$

where α_j depends implicitly on $\bar{\alpha}$ through Eq. 16. Similarly, the total sampled clone count can be explicitly expressed as

$$C^{\mathrm{s}}(\bar{\alpha},\lambda,\eta)=Q\sum_{j=1}^{J}\frac{b_j}{C_\star}\left[1-\left(\frac{2\bar{\alpha}}{\eta\lambda+2\bar{\alpha}}\right)^{2\alpha_j}\right]. \tag{S14}$$

3 SMALL AVERAGE IMMIGRATION RATE

Here, we show that if the support of $\pi_\alpha(\alpha)$ is sufficiently small, the exponential term in Eq. 11 $(\,\cdot\,)^{\alpha/r}\sim 1$, and the product term $\sim(\alpha/r)(k-1)!$. While α is summed or integrated over, for reasonable distributions $\pi_\alpha(\alpha)$, the lowest few rates contribute the most and the average of a function over $\pi_\alpha(\alpha)$ can be replaced by its value evaluated at the small average value $\bar{\alpha}$. Even though for r is integrated over $(0,1)$ for $w=1$, and the region near 0^+ would lead to a large α/r, the contribution from $c_k^{\mathrm{s}}(\alpha,r,\lambda,\eta)$ is also small near $r=0$. We have numerically checked that for all cases of $\bar{\alpha}\ll 1/2$, c_k^{s} can be approximated by

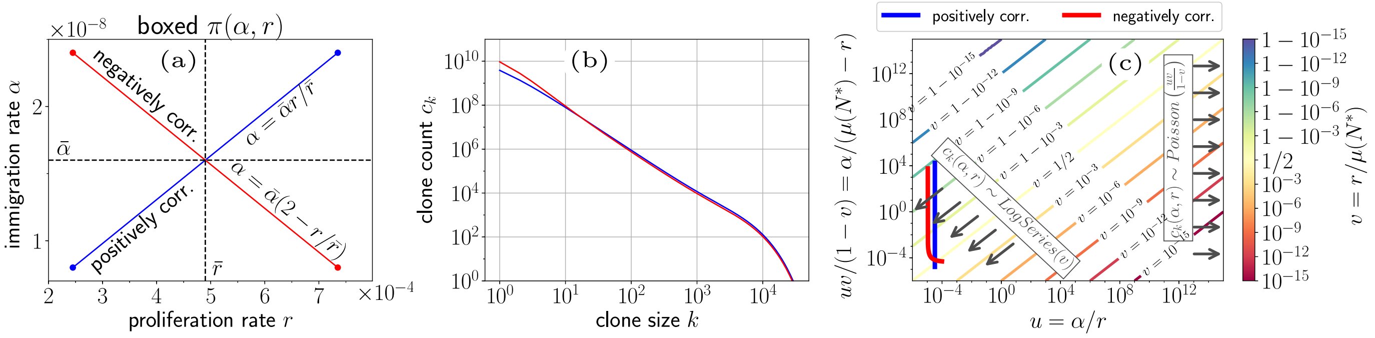

Figure S1. Positively and negatively correlated $\pi(\alpha, r)$. (a) For $\bar{r}/2 \le r \le 2\bar{r}$, we consider $\pi(\alpha, r)$ distributions with positively and negatively correlated α and r (Eqs. S18). (b) Mean sampled clone counts corresponding to positively and negatively correlated $\pi(\alpha, r)$ show negligible differences. (c) "Line integrals" of the positively and negatively correlated distributions $\pi(\alpha, r)$ in the $uv/(1-v)$-u diagram. Clones counts predicted by such $\pi(\alpha, r)$ follow log-series distributions, similar to those of a neutral model.

$$c_k^{\mathrm{s}}(\alpha, r, \mu^*, \eta) \approx \frac{\alpha Q}{rk}\left(\frac{\eta r/\mu^*}{1-(1-\eta)r/\mu^*}\right)^k. \tag{S15}$$

Thus, for general w, f_k^{s} in Eq. 19 can be approximated by

$$f_k^{\mathrm{s}}(\bar{\alpha}, \lambda, w, \eta) \equiv \frac{kc_k^{\mathrm{s}}}{Q\eta\lambda} = \frac{\bar{\alpha}}{\eta\lambda w}\int_{\frac{1}{2}-\frac{w}{2}}^{\frac{1}{2}+\frac{w}{2}}\left(\frac{\eta r/\mu^*}{1-(1-\eta)r/\mu^*}\right)^k \frac{\mathrm{d}r}{r}, \tag{S16}$$

where $\lambda \equiv N^*/Q$ and μ^* is given by

$$\mu^* = \frac{\left(\frac{1}{2}+\frac{w}{2}\right)e^{\lambda w/\bar{\alpha}} - \left(\frac{1}{2}-\frac{w}{2}\right)}{e^{\lambda w/\bar{\alpha}} - 1}. \tag{S17}$$

Since only $\bar{\alpha}$ appears in Eqs. S16 and S17, the irrelevance of the shape of $\pi_\alpha(\alpha)$ is apparent. We have explicitly shown that for small $\bar{\alpha} \ll 1/2$, the approximations in Eqs. S15 and S16 are quantitatively accurate. These simpler forms expedite our numerical analysis and fitting to data using Eq. 20.

4 CORRELATED IMMIGRATION AND PROLIFERATION RATES

Hitherto, we have considered independent immigration and proliferation, and assumed a factorisable rate distribution $\pi(\alpha, r) = \pi_\alpha(\alpha)\pi_r(r)$. However, immigration and proliferation rates may be correlated for certain clones. For example, a frequent realization of V(D)J recombination may also result in a TCR that is more likely to be activated for proliferation. In this case, α would be positively correlated with r. In Fig. S1 we use dimensional rates and consider the effect of correlated $\pi(\alpha, r)$. For $\bar{r}/2 \le r \le 2\bar{r}$, we considered normalized, positively/negatively correlated box distributions as shown in Fig S1(a):

$$\text{Positively correlated}: \quad \pi(\alpha, r) = \frac{1}{\bar{r}}\delta\left(\alpha - \frac{\bar{\alpha}}{\bar{r}}r\right),$$
$$\text{Negatively correlated}: \quad \pi(\alpha, r) = \frac{1}{\bar{r}}\delta\left(\alpha - \bar{\alpha}\left(2 - \frac{r}{\bar{r}}\right)\right). \tag{S18}$$

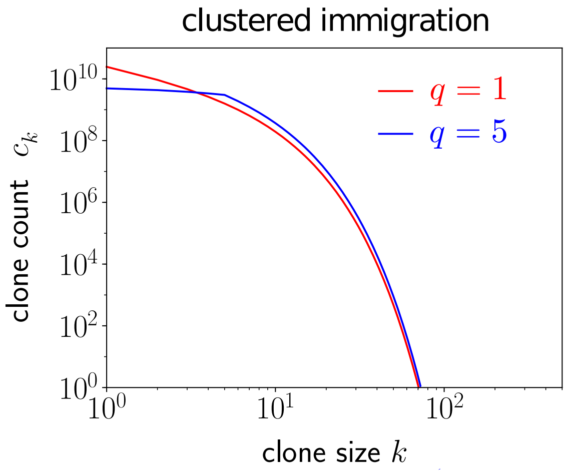

Figure S2. Clustered immigration in a neutral model. Comparison of clone abundances for a $q = 1$ and $q = 5$ models. The difference between the two predicted mean clone counts arise for $k \lesssim q$. Even after sampling, clone counts predicted under clustered immigration ($q > 1$) yields a more slowly decreasing $c_k^{\rm s}$ for small $k \lesssim q$.

Within our mean field model, these positively and negatively correlated distributions $\pi(\alpha, r)$ result in very similar expected clone abundance distributions c_k (Fig S1(b)). This insensitivity to correlations between immigration and proliferation can be qualitatively understood by considering the "line integral" over dominant paths of $\pi(\alpha, r)$ in the $uv/(1-v) = \alpha/(\mu^* - r)$ *vs.* $u = \alpha/r$ diagram, as shown in Fig. S1(c). Both line integrals remain in the log-series distribution regime, indicating that the clone abundance distributions are qualitatively similar to those predicted by a model with proliferation heterogeneity alone.

5 MEAN CLONE COUNTS FOR CLUSTERED IMMIGRATION

We explore how clustered emigration from the thymus affects the mean clone count c_k. Suppose that q cells of the same clone (TCR nucleotide or amino acid sequence) are simultaneously exported by the thymus. The equation for the mean clone count c_k becomes

$$\frac{{\rm d}c_k}{{\rm d}t} = \sum_q \alpha_q \left[c_{k-q} - c_k\right] + r\left[(k-1)c_{k-1} - kc_k\right] + \mu(N)\left[(k+1)c_{k+1} - kc_k\right]. \qquad \text{(S19)}$$

This equation does not admit a simple analytic solution so we numerically solved the equation assuming $\alpha_q = \alpha_5 \mathbb{1}(q, 5)$ and $Q = 10^{11}$. Fig. S2 compares the shapes of c_k for single cell immigration ($q = 1$) and simultaneous multicell immigration $q = 5$. In general, for $q > 1$, c_k, and ultimately $c_k^{\rm s}$ and $f_k^{\rm s}$ are flatter up to $k \approx q$, making the clone counts more sharply kink downwards near q. Thus, as can be seen from Fig. 9(a,b), we can reasonably conclude that some level of paired immigration would provide even better fits to the data at appropriately small values of λ, especially for the first few k-points.
